# Interventions to reduce empathy-based stress and enhance compassionate care in mental health wards: a systematic review

**DOI:** 10.1186/s12913-025-13861-9

**Published:** 2025-12-22

**Authors:** Lucy Maddox, Kevin Teoh, Saffron Baldoza, Lucy Clarkson, Rhiannon Evans

**Affiliations:** 1 Department of Psychology, Claverton Down, University of Bath, Bath , BA2 7AY UK; 2 Birkbeck College London, Malet Street, London, WC1E UK; 3 University of Cardiff, Park Place, CF10 3AT Cardiff, UK; 4 University of Cardiff, DECIPHer sbarc|spark, Maindy Road, Cathays, Cardiff, CF24 4HQ UK

**Keywords:** Empathy-based stress, Compassion, Burnout, Secondary trauma, Patient care, Staff wellbeing, Mental health, Inpatient, Wards

## Abstract

**Background:**

Mental health wards are an important healthcare context with the potential to positively impact patient trajectories. Compassionate care in these wards is important, and can be impacted by staff levels of empathy-based stress (compassion fatigue, burnout and secondary trauma). It is important to consider the evidence-base for mental health ward interventions to improve compassionate care for patients and to reduce empathy-based stress for staff.

**Methods:**

A systematic review was conducted of robust evaluations of mental health ward interventions designed to improve compassionate care and/or reduce staff empathy-based stress, with the aim of synthesising interventional evidence on these interventions’ effectiveness, implementation and acceptability. Programme theory papers, outcome evaluations (RCTs and non-RCTs), economic evaluations and process evaluations were included. A meta-integration of intervention content, effectiveness and influence of contextual factors on implementation and acceptability was performed.

**Results:**

18 eligible study reports of 11 interventions were identified. Interventions were multi-level, and aimed to increase staff resources rather than decrease staff demands. Staff training interventions were most evaluated, with mixed evidence for effectiveness. Other approaches included changes to ward approach, environment, use of participatory action research methods and peer-review programmes. There was no clear evidence for a particular intervention type. Two interventions showed evidence of iatrogenic harm. Equity harms and economic effects were not well-evaluated. Mechanisms of change were under-theorised and lacked clear logic models. Patient and public involvement was sporadic.

**Conclusions:**

Current interventions are being offered without a clear evidence-base or guiding model, and risk harming staff. Multi-level interventions using clearer logic models which tackle both job demands and resources are recommended. A model of implementation factors which may help interventions to succeed is proposed. More high-quality controlled intervention studies, considering contextual and process factors, and incorporating co-production, are needed, especially given the risk of iatrogenic harm.

**Supplementary Information:**

The online version contains supplementary material available at 10.1186/s12913-025-13861-9.

## Introduction

Compassionate care involves empathising and seeking to alleviate suffering through practical caregiving action [1]. Compassion is an important principle of healthcare and part of the National Health Service (NHS) constitution [2] and the World Health Organisation (WHO) values statement. Patients report compassion to be vital to good care [3]. The Francis report, written in the wake of patient harm in mid-Staffordshire NHS Trust, highlighted the devastating, and sometimes fatal, effects of its lack [4].

There are a range of risk factors for poor compassionate care. One of these is staff experience of empathy-based stress, which has been shown to impact care in a range of professions and settings [5]. Empathy-based stress is an umbrella term encompassing compassion fatigue, vicarious or secondary trauma, and burnout [6]. Compassion fatigue is profound emotional and physical exhaustion causing caregivers to feel less able to care [7]. This is also known as empathy fatigue. Vicarious or secondary trauma is the experience of post-traumatic reactions to another person’s traumatic event [8]. Burnout is the triad of emotional exhaustion, depersonalisation and decreased personal accomplishment in response to work stress [9]. A separate but related concept is that of moral injury, which encompasses negative reactions such as guilt, shame, or anger, related to violation or suppression of deeply held moral values [10]. Not only is empathy-based stress potentially bad for patient care, it is also bad for staff wellbeing [11], and for healthcare services, as it increases staff absenteeism and reduces staff retention [12].

Mental health wards are a good place to study empathy-based stress and compassionate care. Mental health professionals in general are at risk of empathy-based stress [13], with an estimated prevalence of 40% for emotional exhaustion [14]. Mental health ward nurses are at increased risk of burnout compared to other ward nurses [15] and mental health ward staff are exposed to significant stressors of violence, patient trauma, self-harming behaviours, and use of Mental Health Act legislation to impose care (e.g. using sections of the Mental Health Act to compel assessment and/or treatment) [16]. Compassionate care in mental health wards is important: responsive and compassionate care during an admission can encourage recovery, whereas its lack can result in inadvertent harm through treatment (iatrogenic harm) [17].

Existing reviews synthesise the evidence-base for interventions for some components of empathy-based stress in general healthcare settings [18–20], but to date there is no systematic review synthesising evaluation evidence for compassionate care or empathy-based stress programmes delivered on mental health wards. It is important to conduct a review of effectiveness to understand which programme theories exist and which work best for whom, in this context. Programme theories, also referred to sometimes as logic models or theories of change, are descriptions of how an intervention is supposed to bring about change, with clear links between the components of the intervention, the implementation strategies, and the expected outputs and outcomes.

This review will classify and describe interventions in relation to two frameworks which describe levels at which interventions work: Bronfenbrenner’s socio-ecological model [21] and the IGLOO model [22]. Both models were used, as although there are overlaps, there are also differences, and they stem from different theoretical foundations and provide different perspectives (systemic versus organisational). PPI consultants valued being able to look at the different levels together.

Programme theories in existing interventions can be categorised as operating across multiple socio-ecological domains (individual, interpersonal, institutional, community, societal) [21], or IGLOO levels (individual, group, leader, organisation, overarching context) [22]. Historically, interventions have focussed on individual-level cognitive and behavioural approaches to enhance coping and reduce maintaining factors (variables which exacerbate or prolong the problem), despite recognition of organisational contributors to empathy-based stress [23]. More recently, attention has shifted towards organisational-level interventions targeting systemic influences on individuals, largely informed by job demands-resources theory [24].

Job demands-resources theory proposes that job satisfaction and burnout relate to the balance between the demands a job makes upon someone, and the resources available to undertake these [24]. High demands may not cause burnout if sufficient resources are provided, whereas even low-demand roles can be stressful when resources are inadequate. Organisational-based interventions might try to reduce demands on staff (e.g. lowering expectations of the number of 1:1 sessions offered to patients per shift) or increase available resources (e.g. allocating dedicated time for staff-patient sessions). This review will examine the systems level at which interventions operate, to determine whether they target individual practices, organisational contexts, or both.

In accordance with best practice methodological guidance on intervention development and evaluation and systematic review, it is also important to review how contextual factors influence intervention functioning, implementation and acceptability [25, 26]. Mental health wards have a constellation of unique contextual challenges, including specific aids and barriers to compassionate care [27], e.g. amount of resource provision and adequacy of communication, that need to be understood to avoid the tendency to create interventions which are “ad hoc and based on anecdotal understandings” [16]. A state of the field evidence synthesis could inform development, adaptation and implementation of effective compassionate care and/or empathy-stress interventions in mental health wards [28]. Since literature in healthcare staff wellbeing has proliferated since the covid-19 pandemic [29], this literature review will be particularly timely.

In addition to these theoretical rationales, patient and public involvement (PPI) was integral to developing the systematic review aims, scope and reporting.

### Aim and questions

This systematic review synthesises international evidence on the effectiveness, implementation and acceptability of interventions targeting empathy-based stress and/or compassionate care in mental health wards. The review was conducted in two phases.

Phase 1 mapped existing interventions and addressed the following question:

Q1. What are the components, mechanisms of change and outcomes evaluated in interventions aiming to prevent and/or reduce staff empathy-based stress, or to enhance compassionate care, in mental health wards?

Phase 2 synthesised evidence on effectiveness, implementation and acceptability, addressing:

Q2. What is the effectiveness of these interventions, including potential equity harms and economic effects?

Q3. How do contextual factors (e.g. settings and people delivering or receiving interventions) impact on intervention implementation and acceptability?

## Method

### Protocol and registration

The protocol was registered with the International Prospective Register of Systematic Reviews (PROSPERO, registration number CRD42023414372) and reported with reference to PRISMA-P systematic review guidelines.

### Patient and public involvement research advisory group

A project Patient and Public Involvement Research Advisory Group (PPIRAG) was comprised of eight participants: mental health ward staff, managers, commissioners, service users and parents/carers. The group was facilitated by the lead author (a consultant clinical psychologist) and a PPI consultant with experience of mental health ward care (author LC). The group met six-monthly. All members helped delineate review scope and one authored this paper (in addition to the PPI consultant). The GRIPP2 Short Form Checklist [30] in Table S0 reports group involvement.

### Information sources

Study reports were identified from six bibliographic databases: MedLine, Embase, PsychInfo, CINAHL, Business Source Complete, and the Cochrane Central Register of Controlled Trials. Grey literature was consulted through websites of the National Institute of Clinical Excellence (NICE), NHS England, National Institute of Health Research (NIHR), King’s Fund, UK government websites. Reference lists of included papers were hand-searched. Initial searches occurred between 7/3/2023 to 19/5/2023 and were updated on 12/9/2024 and 13/9/2024.

### Eligibility criteria

Review parameters were defined in accordance with the PICOS mnemonic (Table S1).

#### Inclusion

Interventions were included if they aimed to disrupt existing system practices or introduce new knowledge or experience at an individual level, with the aim of reducing compassion fatigue, secondary trauma or burnout, or improving compassionate care. Interventions could be mono- or multi-component and target any of the following domains: individual, team/interpersonal, leadership, organisational, and policy/legal. Compassionate care was defined to include the widely used construct of the therapeutic relationship between healthcare professional and patient. A broad definition of empathy-based stress, encompassing compassion fatigue, burnout, secondary trauma and moral injury, was used due to substantial conceptual overlap [6]. A range of study designs were included depending on the research question: outcome evaluations with a control group (Randomised Controlled Trials (RCTs) and non-randomised studies), programme theory papers relating to eligible outcome evaluations, process evaluations and economic evaluations of relevant interventions. Interventions could target mental health ward patients or staff.

#### Exclusion

Studies were excluded if they focused on pharmacological interventions, evaluated impacts on patient seclusion or violence, were outcome evaluations without a control condition (either usual care or active control) or were systematic reviews.

### Search strategy

A search strategy was developed and tested in Medline before adaptation to each database. Search terms mapped on the PICOS mnemonic and included a combination of MESH headings and free text terms (Table S2). There were no language or date restrictions. The search deviated from the original protocol as one database was inaccessible (ASSIA).

### Selection process

Study reports were downloaded, combined, uploaded into Covidence and de-duplicated. A screening tool was developed and calibrated by four reviewers screening a subsample of twenty titles and abstracts. All study reports were independently double screened by a team of six raters based on titles and abstract (LM, RL, KK, AO, EE, PG). Full study reports were screened by the first author and one other rater, independently (EE, RL, KK, AO). Queries were discussed. Where there were unresolvable conflicts or insufficient information, study reports progressed to the next stage of selection. At the final stage a third reviewer’s opinion resolved conflicts (RE). Figure S0 depicts the Preferred Reporting Items for Systematic Reviews and Meta-Analyses (PRISMA) flow diagram (supplementary tables).

### Data collection process

Data extraction templates in Microsoft Word were piloted by four reviewers with 20% of reports. One reviewer extracted data and a second checked it. Missing data were identified through personal correspondence with authors where possible.

### Data items

Data were extracted on study characteristics, intervention characteristics, and review questions. For all interventions, TIDieR Checklist items [31] were collected, along with the intervention level according to the socioecological model [21] and the IGLOO model [22] and whether the intervention targeted job demands or resources [24]. PPI involvement in intervention development was recorded, and intervention quality was assessed using three criteria: reference to theory, facilitator training and measurement of intervention adherence [32].

For theory study reports, data were extracted on theory name and description, key authors, mechanisms of change, related interventions and the presence of a logic model and empirical testing. For outcome evaluations measurements of primary and secondary outcomes were collected, including follow-up outcomes at population and subgroup levels, drop out, results and recommendations, data completeness, baseline differences, adjustment for differences, control of confounding, reporting of adverse events and equity harms using the PROGRESS-PLUS framework [33]. For RCTs, additional data on sequence generation, allocation concealment and blinding were extracted. For process evaluations context, acceptability and implementation data was collected using the Context and Implementation of Complex Interventions Framework (CICI) [34]. Missing data were reported and incorporated into the risk of bias assessment.

### Quality and risk of bias in individual studies

Quality and risk of bias of individual studies was evaluated using relevant quality assessment tools: ROB2 for randomised controlled trials (RCTs) [35], ROBINS-I for non-RCTs [36], Drummond checklist for economic evaluations [37], and two measures designed for quality assessment of theory and process papers respectively [32, 38]. All studies were independently quality assessed by two researchers (LM, CB) and any discrepancies discussed and resolved.

### Effect measure

Effect measures were collected for outcome evaluations. Where standardised effect sizes were not reported, mean differences, t-scores, and F-scores were.

### Synthesis methods

Following meta-integration principles [39], this review employed a convergent synthesis design [40], in which qualitative and quantitative data were first analysed separately and then integrated. This approach enables a comprehensive understanding by allowing an in-depth analysis of each data type, followed by comparison to identify patterns, contradictions and agreements that may otherwise remain hidden. Data tables are presented visually in the results section and in the supplementary materials. Recommendations were developed from the findings, with an initial recommendation drafted for each key finding (LM), and refined through discussion among the authors, considering evidence gaps, methodological limitations and the wider policy and practice context.

## Results

### Study characteristics

Of 3705 abstracts screened, 18 study reports were included, describing 11 interventions. Studies were conducted in various Western countries: five each from the UK [41–45], and Canada [46–50], one each in the USA [51], Norway [52], Australia [53], Switzerland [54], Sweden [55] and Spain [56]. Two reports related to more than one country: Canada and Scotland [57] and Australia and UK [58]. Studies were published between 1976 and 2019. There were two programme theory study reports [46, 49], 11 outcome evaluations, of which seven were RCTs and four non-RCTs [41–43, 45, 51–56, 58], one economic evaluation [47] and four process evaluations [44, 46, 48, 50, 57]. Table S3 shows study reports, study types and related interventions. Studies were in general (adult) mental health wards [42, 45, 51–54, 56, 58] or forensic wards [41, 43, 44], with one study recruiting from both [55].

#### Study quality and risk of bias

Programme theory papers [46, 49] were both assessed as high quality, despite a lack of logic model. For outcome evaluations, RCTs all had either some concerns or high concerns (Figure S1). For the two with high concerns, they deviated from the intended intervention [51], or had randomisation flaws [58]. One study nearly met all criteria for low risk of bias, only having some concerns on selection of reported result [45]. Non-RCTs all had either moderate or serious risk of bias (Figure S2). Three studies had serious risk of bias [53, 55, 56] with serious or moderate concerns across multiple domains. One study had moderate risk of bias due to moderate concerns about confounding and selection of the reported result [52]. The single economic evaluation received an average rating [47]. Process evaluations were mixed. Two scored medium on both domains of reliability/trustworthiness and usefulness [44, 48], two were rated low for reliability (due to lack of explanation of methods) and medium for usefulness [50, 57].

#### Quality of intervention

The 11 interventions were of mixed quality. All referred to a theory, although without logic models. The person delivering the intervention had adequate training in most cases (bar two). Only two studies checked intervention integrity, meaning that it is hard to know if an intervention has failed due to an implementation error or a lack of efficacy (Table S4 details further).

### Intervention components, mechanisms of change and outcomes

Components, outcomes and mechanisms of change are described in Table S5 and summarised in Table 1.

**Table 1:** Summary table of interventions and effectiveness

Intervention name and references	Brief description	Level		Demands or resources	Quality score (out of 3)	PPI score (out of 3)	Effective?
		SE	IGLOO				
Behaviour Modification Skills Training for Staff [58]	4 day training (over 4 week period) in staff behaviour modification skills aims to reduce burnout through improved self-efficacy and modification of attitudes (empathy-based stress)	Individual, interpersonal Community	Individual, Group	Resources	1	0	No
Micro-counselling training for staff [51]	6 week training in micro-counselling skills (both supervised and un-supervised) aims to improve therapeutic relationship between staff and patients (compassionate care)	Individual, intrapersonal community	Individual, Group	Resources	2	0	Mixed
Mindfulness Based Stress Reduction Staff Training and Affect Consciousness Staff Training [52]	8 week training in either MBSR or AC aims to improve reflective capacity in staff and improve compassionate care (compassionate care)	Individual, interpersonal, community	Individual Group	Resources	2	0	Yes
Model of Integrated Care in Mental Health (change to ward approach) [54]	Change in ward practice to allow relationships in community and inpatient care to sustain with aim that improved continuity of care will improve compassionate care for the patient. Followed up a year post-discharge. (compassionate care)	Interpersonal, institutional	Group organisation	Resources	2	0	No
Participatory action research into improving therapeutic relationship [56]	10 month participatory action research group with nurses on the ward to explore therapeutic relationship. Led to adoption of reflective groups, protected patient time and journal article reading (compassionate care)	Individual, interpersonal, community	Individual, group	Resources	1	1	Mixed
Peer-Led Quality Improvement Network [41, 44]	Several weeks of self-review against consensus standards followed by a day of independent peer assessment and feedback with aim of improving care quality (compassionate care)	Interpersonal, institutional, community,	Group, leadership, organisational overarching	Resources	2	0	No
Psychosocial Ways of Working [43]	20 day training in psychosocial ways of working with aim that staff having better knowledge and attitudes about treatment for chronic mental illness would lead to a greater sense of efficacy and lower burnout (empathy-based stress)	Individual, intrapersonal, community	Individual Group	Resources	2	0	Yes
Rebuilding of 2 wards [53]	Relocation to purpose-built wards with aim that improvement to ward environment will influence staff burnout by improving patient and staff wellbeing in the setting (improved opportunities for patient-staff interaction and nicer working environment) (empathy-based stress)	Institutional	Organisational	Resources	1	0	No
Steps towards recovery [55]	Change in ward approach to involve behavioural activation for patients, using combination of staff training, resources, coaching and change to ward structure of the day, with aim to improve both patient care and staff burnout through improvement of self-efficacy (compassionate care and empathy-based stress)	Individual, interpersonal community	Individual, leadership, group	Resources	2	2	No
Therapeutic Group Training for Staff [42, 45)	6 months staff training in how to facilitate therapeutic groups with aim to improve patient experience of care and staff burnout (via staff morale) (compassionate care and empathy-based stress)	Individual, intrapersonal,	Individual, group	Resources	2	3	Mixed
Transitional Discharge Model [46–50, 57]	Change in ward approach to create a continuous relationship with staff between inpatient and community services, and provision of a peer support network, with the aim of improving compassionate care (compassionate care)	Individual Interpersonal, Institutional, Community	Individual, leadership, organisational	Resources	3	0	No

KEY: SE = socioecological model, IGLOO = Individual, Group, Leadership, Organisation, overarching Context, PPI,=Patient and public involvement, MBSR = Mindfulness based stress reduction, AC = Affect Consciousness

### Classification and description of intervention components

#### Levels of intervention and relation to job demands and resources

Interventions were all multi-level, but intervened at the individual, intrapersonal and community levels, more frequently than the leadership, organisational or overarching context/policy levels. Specifically, in relation to the socioecological model [21], eight interventions described an individual level intervention component [42, 43, 45, 49–52, 55, 56, 58] ten described intrapersonal components [41–45, 49–52, 54–56, 58] four described institutional components [41, 49, 50, 53, 54, 58] eight described community components [41, 43, 49–52, 55, 56, 58] and none described components relating to the level of public policy. In relation to the IGLOO model [22], eight interventions described individual level intervention components [42, 43, 45, 49–52, 55, 56, 58], nine described group level components [41–43, 45, 51, 52, 54–56, 58], three had leadership level components [41, 49, 50, 55] four had organisational level components [41, 49, 50, 53, 54], and one had components relating to overarching context [41].

All interventions aimed to improve staff resources, with none reducing staff demands [24]. Table 2 maps types of demands or resource addressed and at which level. Targeted individual resources related mostly to clinical skills, rather than staff psychoeducation and self-care; intrapersonal skills related to staff-patient interactions rather than staff-staff interactions; and leadership interventions did not address leadership or management skills or knowledge frameworks. The relatively few institutional and organisational interventions concerned the physical environment or a new patient focus, rather than system-level changes which affecting staff wellbeing (e.g. staffing rotas or use of bank/agency staff).

**Table 2:** Demands and resources targeted across organisational levels

Socioecological level	IGLOO level	Demands	Resources
Individual	Individual		Improved clinical skills Improved clinical knowledge Improved reflective capacity
Intrapersonal			Focus on therapeutic relationship Training in micro-skills relating to patient interactions Training in small staff groups encourages relationships
Community	Group		Increased team continuity Team learning together Peer review opportunities
	Leadership		Leadership emphasis on different model of care
Institution	Organisation		New ward environment Patient-focus emphasised
Societal/Policy	Overarching Context		Connections to a wider peer network

#### Patient and public involvement

PPI was reported in four studies but only one study used it in all domains of intervention design, delivery and evaluation (see Table S6).

#### Intervention content

**Fig. 1:**
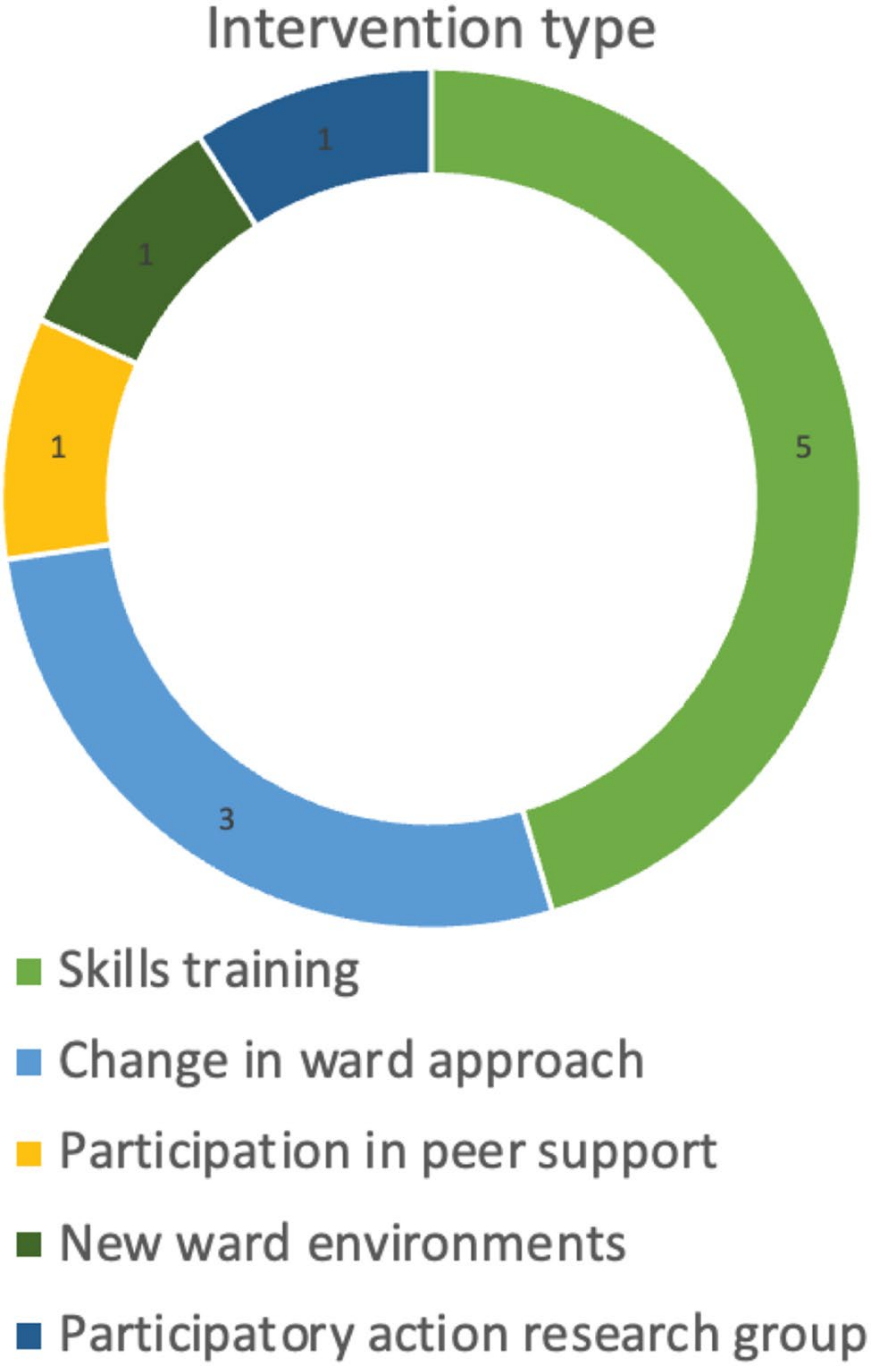
Main intervention component type

Five interventions involved staff skills training: behaviour modification [58], micro-counselling [51], mindfulness based stress reduction and affect consciousness [52], psychosocial ways of working [43] and facilitating therapeutic groups [42, 45]. Three targeted ward approach: the Model of Integrated Care in Mental Health [54] emphasised continuity between inpatient and community care, Steps Towards Recovery [55] introduced a behavioural activation-based ward programme, and Transitional Discharge Model [49, 50] combined continuity of inpatient and community care with patient peer support. Additional interventions involved a professional peer support network [41, 44], participatory action research on therapeutic relationships [56], and new ward environments [53] (Fig. 1).

#### Mechanisms of change

All interventions had a rationale, but mechanisms of change were under-theorised. Five hypothesised that increasing staff therapeutic skills would improve staff satisfaction, reduce burnout and/or improve care [42, 43, 45, 51, 55, 58]. Two theorised that improved continuity of care would improve compassionate care [49, 50, 54]. One targeted staff reflection and emotional regulation [52] to support staff coping and patient care, and one theorised that peer-review would improve care quality via social learning and reduced burnout [41]. One provoked staff to consider improvements in therapeutic relationships [56]. Mechanisms of change were seldom evaluated, with process evaluations available for only two of the eleven interventions: Transitional Discharge Model [46, 48, 57] and Peer-led Quality Improvement Network [44].

#### Targeted outcomes

Five of the eleven interventions measured effects on compassionate care [49–52, 54, 56] two on empathy-based stress [43, 58] and four on both [41, 42, 45, 53, 55] (Table 3).

**Table 3:** Targeted outcomes

Compassionate care	Empathy-based stress	Both
Mindfulness-based stress reduction & affect consciousness training	Behaviour modification skills training	Therapeutic group training
Micro-counselling training	Psychosocial Ways of Working training	Peer-led quality improvement network
Model of Integrated Care ward approach		Rebuilding two wards
Transitional Discharge Model ward approach		Steps Towards Recovery (behavioural activation-based ward approach)
Participatory Action Research into therapeutic relationship		

Compassionate care interventions used diverse measures, whilst empathy-based stress interventions were more consistent (Tables S7 and S8). Among five compassionate care and four dual interventions, three measured therapeutic relationships [51, 54, 56] using the Ideal Therapeutic Relationship Scale [51], the Scale to Assess Therapeutic Relationships (STAR-D) [54], and the Working Alliance Inventory [56]. One measured patient perceptions of care, using the Views On Inpatient Care (VOICE) measure [45], another assessed staff support of patients using the support sub-section of the Ward Atmosphere Scale [52], and one used observations of patient-staff interactions [53]. Quality of patient care was measured by the Quality of Psychiatric Care – Inpatient Staff Scale (QPC-IPS) [55] and patient satisfaction by a modified version of the Patient Satisfaction Questionnaire (PSQ) [41]. One economic evaluation used patient Quality of Life, in line with economic evaluation practice. In contrast, the two empathy-based stress interventions and four dual interventions, focussed on burnout using the Maslach Burnout Inventory (MBI) [41–43, 53, 55, 58].

### Intervention effectiveness, equity harms and economic effects

Detailed effectiveness data are provided in tables S7 and S8, and summary findings presented in Table 1.

#### Effectiveness of interventions targeting compassionate care

Of the nine interventions targeting compassionate care, four showed some effectiveness. Two RCTs found tentative evidence: a six-month staff training in therapeutic group provision [45] found particular benefit for patients detained under the Mental Health Act despite no overall effect on patient care scores, though there were some concerns about selective reporting; and a six-week staff micro-counselling training [51], showed improvement but was rated high risk for bias due to deviation from the intended intervention. Two non-RCTs also indicated improvements: an eight-week staff mindfulness-based stress reduction training (moderate risk of bias); [52], and a ten-month participatory action research programme, (high risk of bias) [56]. Only the therapeutic group training study reported an effect size [45] (standardised 0.18).

#### Effectiveness of interventions targeting empathy based stress

Of the two empathy-based stress interventions and the four targeting both empathy-based stress and compassionate care, only one showed some effectiveness. An RCT [43] of staff training in psychosocial ways of working reported significant reductions in all three MBI burnout subscales, though the sample was small with some risk of bias.

#### Direct harms

Some iatrogenic effects were observed. Staff burnout increased in the peer-led quality improvement network [41]; and after moving into rebuilt wards [53]. One MBI subscale decreased in control wards as compared to wards where staff received behavioural activation training [55]. The first intervention had some risk of bias, while the latter two were rated as having serious risk.

#### Equity harms

No study reported all PROGRESS-PLUS characteristics; age, gender, sex and role were most common (Table S9). Only one study conducted a subgroup analysis to assess equity harms, finding none [42].

#### Economic effectiveness

Economic evaluation was rare. The Transitional Discharge Model [47] showed no significant improvements in quality of life or reduction in health and social care costs post-discharge. An RCT of staff therapeutic group facilitation training [45] calculated costs of approximately £10 per patient per week, versus a non-significant £12 decrease in resource allocation towards patient perceived meaningful contacts.

### The impact of contextual factors on intervention implementation and acceptability

Process evaluations from four studies [44, 46, 48, 57] and qualitative feedback from outcome evaluations were analysed (Table S10). Although data were insufficient for full thematic analysis, four themes were identified: available resources, staff attitudes to change, roles and relationships and stakeholder involvement in intervention design.

#### Available resources

Implementation and staff acceptability improved when supportive resources were available, whether related to organisational structures, time or personnel. The Transitional Discharge Model [57] was implemented more effectively in Canada than in Scotland, due to better on-ward resources and existing infrastructures, i.e. supportive documentation systems, champions, and accessible training [57]. Organisational support was described as key [57]. Conversely, a UK-based participatory action research intervention lacked support, and had high staff dropout rate (approx. 50%) [56]. Shorter interventions were generally more acceptable to staff [58], although six weeks of micro-counselling training [51] and four weeks of behaviour modification skills training [58] were considered too short. Time spent on interventions outside of training sessions helped when supported by a supervisor [42, 45] but had less uptake if framed as “homework”, e.g. under 25% of staff practiced mindfulness for the recommended 3 times weekly [52]. Human resources were crucial, with turnover and shortages hindering delivery [41, 48, 55], although one study noted positive effects of staff change [48]. Peer-review effectiveness depended on senior and junior staff involvement and strong communication and action planning [44].

#### Staff attitudes to change

Staff readiness for change aided implementation [44], whereas staff feeling under scrutiny was an implementation barrier [41]. Interventions were less successful in contexts of organisational change [48], with staff experiencing educational overload and sometimes refusing participation [48]. These findings highlight the importance of interventions being seen as ongoing, iterative processes within a complex system rather than one-off procedures, to minimise the potential for negative staff attitudes in relation to change or uncertainty.

#### Roles and relationships

Lack of role clarity on wards negatively impacted interventions [48], whether due to uncertainty about responsibilities or over-worked staff having less time to engage. Inter-team relationships were important when interventions involved multiple teams [57], and sharing knowledge among staff was crucial to success [44]. Forchuk et al., (2013) found that addressing team issues prior to intervention implementation improved success.

#### Flexibility, agency and perception of intervention as contextually sensitive

Stakeholder involvement in design, delivery and evaluation was uncommon but appeared to improve face validity. Acceptability increased when staff tailored pre-designed interventions, e.g. choosing training modules [42, 45]. Multi-modal interventions targeting different system levels were considered beneficial [58]. Hospital administrators played important gatekeeping and support roles, and acceptability of the intervention increased when they perceived it to be locally created and solving a local problem [57]. Supervision enhanced training effectiveness, likely through greater tailoring [51]. One study [54] noted that increased flexibility in new ward practices might increase staff demands, although no supporting data were shared. Mechanisms of change in the peer-review intervention [41] involved consultation, ownership and delegation [44]. In the ward rebuilding study [53] lack of staff involvement in design may have reduced acceptability due to unintended consequences, including smaller rooms and increased staff isolation, compounded by limited management flexibility impacting staff wellbeing [53].

### Integration of method-level synthesis

Conclusions about intervention effectiveness remain tentative due to data limitations. There was no clear pattern linking effectiveness to intervention mechanisms, components, or socio-ecological/IGLOO levels. Whilst process data identified four factors that may enhance acceptability and implementation, triangulation is difficult due to the limited quality of effectiveness data and lack of rich process data. Three effective interventions reported perceived flexibility or local tailoring [45, 51, 56] or home practice adaptable to participants’ environments [52], but insufficient data across studies prevents firm conclusions. In addition, limitations in outcome evaluation quality, inconsistent use of PPI in intervention development, and variable measurement of intervention quality hinder assessment of their impact on efficacy.

## Discussion

### Results in context

The review highlights gaps in the current intervention landscape. While all interventions had some theoretical basis, clear logic models or comprehensive programme theories were lacking. Theoretical underpinnings drew on general theories, such as social learning theory [41, 44], or a theory that understanding a psychosocial model improves staff efficacy and reduces burnout [43]). Relying on general theories rather than frameworks specific to empathy-based stress or organisational psychology leaves interventions disconnected from models of empathy-based stress, compassionate care and mental health ward support structures, and unmoored from existing standards of care or staff wellbeing. The absence of clear logic models also complicates assessment of whether interventions are doing what they intended and where they need to be altered.

Interventions all targeted either therapeutic relationship or burnout. None specifically addressed other elements of compassionate care, compassion (or empathy) fatigue or secondary trauma. Debate around the concept of compassion (or empathy) fatigue [59] may contribute to this gap, yet the absence of interventions targeting these well-established problems in mental health wards is notable.

It is striking that all interventions aim to improve resources rather than reduce demands. While reducing demands in this context may seem infeasible to researchers and staff, other healthcare interventions have shown novel ways of reducing job demands and increasing resources which are not represented [60].

Interventions lacked direct mechanisms of change, e.g. targeting burnout by improving staff skills in patient care rather than providing coping strategies. Interpersonal components targeted staff-patient interactions, which are important, but left out staff-staff interaction entirely, despite this being a significant cause of stress [61]. Leadership interventions emphasised new ways of working, rather than harnessing existing skills and structures to support staff management processes [41, 46, 49, 55], despite management quality being an important burnout risk factor [62]. Some of the simplest things seem to have been overlooked, with staff and manager psychoeducation notably absent.

Limitations in outcome evaluation quality make it hard to draw firm conclusions. Some positive effects were noted for four interventions on compassionate care and one on empathy-based stress. No clear evidence emerged for a particular type of intervention component, duration, or mechanism of change. Equity harms were not identified, though they were not systematically assessed. Economic evaluation was robust in only one intervention [47], with one additional RCT presenting costs and benefits [45]. No intervention demonstrated convincing cost-benefit analysis, although Forchuk et al [47]. reflected on this being different had they incorporated length of stay into their economic evaluation.

Importantly, direct harms were reported in two studies [41, 53]. In one, moving wards increased staff burnout, likely due to lack of staff consultation on ward design, poor management response to unintended consequences, and the inherently disruptive nature of relocating. Just as moving house is stressful [63], so is moving workplaces. The second, staff burnout increased following a peer review intervention [41], with some staff feeling scrutinised and burdened by preparatory work. These examples underscore that well-intentioned interventions can increase staff stress.

Four factors enhanced acceptability and implementation: resources, staff readiness, clear roles and good relationships, and flexibility and agency. Flexibility was supported by multi-component interventions allowing staff choice, and supervision enhanced contextual sensitivity. Authors [51, 58] noted that interventions needed to be long enough to have an impact, although two effective interventions lasted only six and eight weeks.

Whilst the evidence-base is expanding, all included studies were published in 2019 or earlier, as more recent research did not meet our search criteria. However, ongoing research may inform future intervention development and provide additional insights into context and implementation. For example, studies from other healthcare settings could offer insights into improving intervention acceptability and take-up on mental health wards [64], and organisational psychology perspectives might be fruitful for clinical researchers [65]. Future review updates could include a broader range of studies to enrich findings.

### Limitations of the evidence-base

There are five main limitations of this evidence-base. First, the lack of clear logic models makes it difficult to be clear on why interventions are being offered in the way that they are, and which components may be most effective. Although theories of empathy-based stress exist [24, 66, 67], they are not consistently experimentally validated, and further research would be beneficial.

Second, high risk of bias in many of non-RCTs and moderate to high risk of bias in RCTs complicates interpretation of efficacy. Some risk reflects older studies conducted before conventions such as pre-registration, while other risk reflects challenges for psychosocial interventions in being able to blind those delivering the intervention to participant condition [68]. However, other concerns related to analysis, control of confounding, contamination and under-implementation. Research in busy ward environments is difficult, but the poor quality of some of the studies limits confidence in the evidence.

Third, a lack of control group in outcome evaluation studies is a problem. From approximately 5000 papers, only 18 met inclusion criteria, often due to a lack of control group. Whilst qualitative studies are valuable, controlled studies are needed to assess interventions, especially given the potential for iatrogenic harm.

Fourth, process evaluation was frequently neglected, limiting the understanding of context implementation quality. When process data were available or authors described implementation and staff experiences, insights into intervention delivery were improved.

Fifth, and finally, no studies researching low and middle income countries (LMIC) were identified, possibly reflecting other resource issues being of a higher concern in these areas, and the lesser prioritisation of mental health burden in LMICs [69].

### Review limitations

This review has three main limitations. First, this review is limited by the quality of the papers within it, which have scant data on context, implementation and acceptability and variable quality. It is hard to know how interventions were implemented, in what ways context interacted, and how acceptable people found the intervention.

Second, incomplete reporting of PROGRESS-PLUS demographic criteria restricts understanding of whether there were equity effects, and whether any health inequalities were perpetuated or exacerbated by interventions.

Third, broad search criteria were adopted to ensure inclusivity and reflect PPI feedback that both empathy-based stress and compassionate care interventions were relevant. Whilst this breadth may reduce focus, it has the benefit of enabling simultaneous consideration of staff and patient quality of life impacts, arguably two sides of the same coin.

**Fig. 2:**
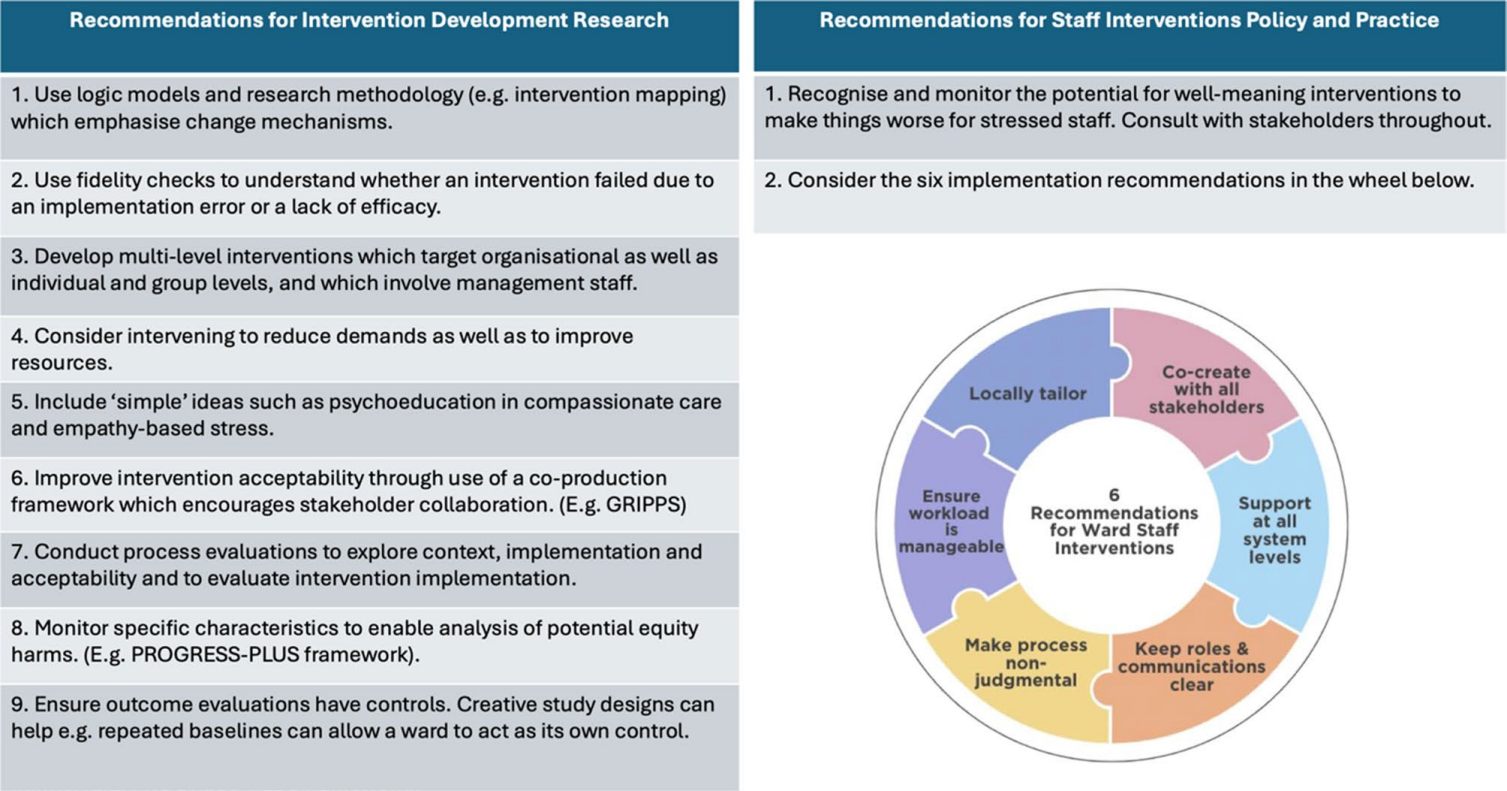
Recommendations for intervention research, policy and practice

Despite these limitations, the review identified nine research and two policy and practice recommendations, developed by the authors including PPI research advisors, and summarised in Fig. 2.

### Research recommendations

We recommend that intervention developers: use clear logic models to design components and verify proposed mechanisms of change before evaluating efficacy; develop multi-level, co-produced interventions that address both job demands and resources; consider ‘simple’ interventions such as psychoeducation; incorporate process evaluation to understand contextual interactions; monitor diversity to ensure equity; and include adequate controls in outcome evaluations.

### Policy and practice recommendations

Staff interventions should be monitored carefully, recognising potential iatrogenic harms. Interventions should be locally tailored, co-created, multi-level and involve clear roles and communications in a non-judgemental style and with a manageable workload. Existing mental health ward standards rarely address these interventions and recommendations may be helpful.

Following these research recommendations would improve the quality of the literature and contribute meaningfully to the work wellbeing evidence base. Policy adherence would protect staff from harm and enable process evaluation data to enhance engagement on mental health wards.

## Conclusions

This review systematically examined interventions to reduce empathy-based stress and improve compassionate care in mental health wards, identifying key areas for improvement. Current interventions often lack a clear evidence-base or guiding model, and can risk harming staff. Iatrogenic harm is an important consideration, especially with overloaded or change-fatigued staff, and robust measurement and thoughtful design are important to avoid this. Further high-quality intervention research in mental health ward settings is needed, considering contextual and process factors, and incorporating co-production. Multi-level interventions guided by clear logic models, addressing organisational factors including both job demands and resources, involving management staff, and employing simple, direct mechanisms of change are recommended. Attention to study and intervention quality is essential to advance this field.

## Supplementary Information

Below is the link to the electronic supplementary material.

Supplementary Material 1

## Data Availability

The data that support the findings of this study are available from the corresponding author, [LM], upon reasonable request.
