# Supplementary material for: Interventions to reduce empathy-based stress and enhance compassionate care in mental health wards: a systematic review
Source: BMC Health Serv Res. 2025 Dec 22;26:117. doi: 10.1186/s12913-025-13861-9 (PMC12837129; doi:10.1186/s12913-025-13861-9)
Supplement: Supplementary file 1 — Supplementary Material 1 [file 12913_2025_13861_MOESM1_ESM.docx]

**Supplementary Figures and Tables**

**Table S0. Reporting patient and Public Involvement in this study using GRIPP2.**

| GRIPP2 Short form item | Description |
| --- | --- |
| Aims: Report the aim of Patient and Public Involvement in the study | Ensure that the voices of stakeholders are included at all stages of the study  To discuss and agree the scope of the review  To discuss what stakeholders would want to see from a systematic review of this nature  To discuss the themes identified in the review to ensure face and content validity and relevance. |
| Methods: Provide a clear description of the methods used for Patient and Public Involvement in the study | The PPIRAG was established by LM at the start of the project. Some members had already been involved in the funding application for the project and others were new. The group represents diverse stakeholder voices. It is facilitated jointly by LM (a clinician and researcher with experience of working on mental health wards) and LC (a co-production consultant with lived experience of mental health wards). The group met six monthly throughout the project’s 5 year span, and also have additional meetings when necessary. |
| Study result outcomes: Report the results of Patient and Public Involvement in the study, including both positive and negative outcomes | The group provided a strong PPI perspective. They critiqued the scope of the review, influencing search terms and scope and extraction criteria. They reviewed the findings from the review and gave thoughts on existing themes and areas they felt were missing.  In particular, the PPI RAG thought that interventions targeting both compassionate care and empathy-based stress should be included in the review, rather than targeting only one of these. They felt that these were flip sides of the same coin and were both important. This significantly influenced the scope of the review.  In addition, the PPI RAG influenced the data that was extracted. The group thought both the IGLOO framework and the Bronfenbrenner socioecological framework had value and thought it would be more comprehensive to include both models in the analysis and they thought it might be helpful to be able to compare to Quality Network of Inpatient Care (QNIC) standards.  There were no significant points of disagreement within the group over what should be included in the review. One theme running throughout the discussions was of the importance of this group of stakeholders getting their voices heard, when often they are not included in research. |
| Discussion and conclusion outcomes: Comment on the extent to which Patient and Public Involvement influenced the study overall. Describe positive and negative effects | The PPI RAG was important in confirming that the review had identified information of importance to stakeholders. This was especially important as PPI was not consistently involved in the studies comprising the review. |
| Reflections/critical perspective: Comment critically on the study, reflecting on the things that went well and those that did not, so others can learn from this experience. | The PPI RAG have been invaluable to the study and influenced decisions throughout. It has been especially helpful to have input from a range of stakeholders from the very beginning of the project, and to have a series of regular meetings to build working relationships. Building in training for all members throughout the project was particularly beneficial.  Over the course of the 5 years of the project, attendance at the research advisory group has always happened, but at times various members of the group could not join for a period of time, due to health concerns or care relating to them or their child. This is part of working with stakeholders, but meant at times the group was smaller. Future research might benefit from having a larger group of stakeholders (perhaps more like 15) to begin with. |

**Table S1: PICOS Criteria for Study Selection**

| Population | Staff in all mental health ward settings |
| --- | --- |
| Intervention | Interventions were included if they aimed either to disrupt existing system practices or to introduce new knowledge or experience at an individual level, with the aim of reducing compassion fatigue, secondary trauma or burnout, or improving compassionate care. Interventions could be mono-component or multi-component and target any of the following domains: individual, team/interpersonal, leadership, organisational, and policy/legal (refs). Studies were excluded if they were of pharmacological interventions (either as a single component intervention or part of a multi-component intervention), or if they were looking at the impact of the intervention on patient seclusion or violence. |
| Comparator | For outcome evaluations there had to be a control (either usual care or active control). |
| Outcome | Compassionate care was defined as including the widely-used construct of therapeutic relationship (between healthcare professional and patient). A wide definition of empathy-based stress which included compassion fatigue, burnout, secondary trauma and moral injury was used due to significant overlap in the concepts (6). Outcomes could be reported subjectively or observed, and both staff-reported and patient-reported. Reporting of adverse events and equity harms were also collected. |
| Study Design | A range of study designs were included depending on the research question addressed: outcome evaluations (RCTs and non-randomised studies), theory description papers relating to a specific intervention which had been assessed by an outcome evaluation, programme theory papers relating to a clear logic model of an intervention, process evaluations relating to relevant interventions, economic evaluations relating to relevant interventions. Systematic reviews were excluded. |

**Table S2. Search strings for all journal searches**

**PUBMED**

| **PICO** | **ID** | **Searches in Title/Abstract** |
| --- | --- | --- |
| Population | #1 | Staff OR worker* OR therapist* OR nurse OR nurses OR nursing OR medic* Or doctor* OR psychiatrist* OR teacher* OR psychologist* OR counsellor* OR counselor* OR social worker* |
|  | #2 | Work/ OR education/ OR nursing/ OR workplace/ OR health personnel/ |
|  | #3 | 1 OR 2 |
|  | #4 | Ward OR inpatient OR tier 4 OR specialist OR acute care OR acute treatment OR admission OR hospitalisation OR hospitalization OR discharge OR PICU OR (psychiatric intensive care unit) |
|  | #5 | Mental health OR psychiatr* OR mental illness OR CAMHS OR (mental adj3 health) OR (mental adj3 illness) OR mental ill health OR (mental adj3 crisis) |
|  | #6 | 3 AND 4 AND 5 |
| Intervention | #7 | Intervention* OR training* OR program* OR workbook* OR leadership OR management OR organisational OR (work adj conditions) OR prevention OR reduction OR improvement OR strategy OR strategies OR policy OR policies OR (mindfulness adj3 training) OR session OR teach* OR course* OR (quality adj3 improvement) OR Balint OR schwarz round OR workload OR caseload OR shift OR supervision OR reflective practice OR environment* OR ward climate OR hospital climate OR team* OR workload OR turnover OR retention OR shift work OR culture |
|  | #8 | Psychotherapy/ OR personnel staffing and scheduling/ |
|  | #9 | 7 OR 8 |
| Outcome | #10 | (compassion adj fatigue) OR burnout OR burn-out OR (secondary adj trauma) OR STS OR (vicarious adj trauma) OR (empath* adj distress) OR staff wellbeing OR moral injury |
|  | #11 | Compassion fatigue/ OR Burnout, psychological/ |
|  | #12 | Compassion* care OR patient care OR therapeutic relationship |
|  | #13 | 10 OR 11 OR 12 |
|  |  | 6 AND 9 AND 13 |

**PSYCHINFO**

| **PICO** | **ID** | **Searches in Title/Abstract** |
| --- | --- | --- |
| Population  #1 Abstract  #2 Title | #1 | Staff OR worker* OR therapist* OR nurse OR nurses OR nursing OR medic* Or doctor* OR psychiatrist* OR teacher* OR psychologist* OR counsellor* OR counselor* OR “social worker*” |
| #3 APA Thesaurus | #2 | Psychiatric Hospital Staff |
|  | #3 | 1 OR 2 OR 3 |
| Title  Abstract | #4 | Ward OR inpatient OR tier 4 OR specialist OR acute care OR “acute treatment” OR admission OR hospitalisation OR hospitalization OR discharge OR PICU OR “psychiatric intensive care unit” |
| Title  Abstract | #5 | Mental health OR psychiatr* OR mental illness OR CAMHS OR “mental NEAR/3 health” OR “mental NEAR/3 illness” OR “mental ill health” OR “mental NEAR/3 crisis” |
|  | #6 | 3 AND 4 AND 5 |
| Intervention | #7 | Intervention* OR training* OR program* OR workbook* OR leadership OR management OR organisational OR “work NEAR/1 conditions” OR prevention OR reduction OR improvement OR strategy OR strategies OR policy OR policies OR “mindfulness NEAR/3 training” OR session OR teach* OR course* OR “quality NEAR/3 improvement” OR Balint OR “schwarz round” OR workload OR caseload OR shift OR supervision OR “reflective practice” OR environment* OR “ward climate” OR “hospital climate” OR team* OR workload OR turnover OR retention OR “shift work” OR culture |
| APA Thesaurus | #8 | Intervention OR Psychotherapy OR Work scheduling |
|  | #9 | 7 OR 8 |
| Outcome | #10 | “compassion fatigue” OR burnout OR burn-out OR “secondary trauma” OR STS OR “vicarious trauma” OR “empath* distress” OR “staff wellbeing” OR “moral injury” |
| APA Thesaurus | #11 | “Compassion fatigue” OR “Burnout” OR “moral injury” OR “patient care” |
|  | #12 | “Compassion* care” OR “patient care” OR “therapeutic relationship” |
|  | #13 | 10 OR 11 OR 12 |
|  |  | 6 AND 9 AND 13 |

**EMBASE**

| **PICO** | **ID** | **Searches in Title/Abstract** |
| --- | --- | --- |
| Population  #1 Abstract  #2 Title | #1 | (Staff OR worker* OR therapist* OR nurse OR nurses OR nursing OR medic* Or doctor* OR psychiatrist* OR teacher* OR psychologist* OR counsellor* OR counselor* OR “social worker*”):ti,ab |
| Emtree | #2 | ‘Health care personnel’/exp OR nursing/exp |
|  | #3 | 1 OR 2 |
| Title  Abstract | #4 | (Ward OR inpatient OR tier 4 OR specialist OR acute care OR “acute treatment” OR admission OR hospitalisation OR hospitalization OR discharge OR PICU OR “psychiatric intensive care unit”):ti,ab |
| Title  Abstract | #5 | (“Mental health” OR psychiatr* OR “mental illness” OR CAMHS OR “mental NEAR/3 health” OR “mental NEAR/3 illness” OR “mental ill health” OR “mental NEAR/3 crisis”):ti,ab |
|  | #6 | 3 AND 4 AND 5 |
| Intervention | #7 | (Intervention* OR training* OR program* OR workbook* OR leadership OR management OR organisational OR “work NEAR/1 conditions” OR prevention OR reduction OR improvement OR strategy OR strategies OR policy OR policies OR “mindfulness NEAR/3 training” OR session OR teach* OR course* OR “quality NEAR/3 improvement” OR Balint OR “schwarz round” OR workload OR caseload OR shift OR supervision OR “reflective practice” OR environment* OR “ward climate” OR “hospital climate” OR team* OR workload OR turnover OR retention OR “shift work” OR culture):ti,ab |
| Emtree | #8 | Psychotherapy/exp OR Work/exp |
|  | #9 | 7 OR 8 |
| Outcome | #10 | (“compassion fatigue” OR burnout OR burn-out OR “secondary trauma” OR STS OR “vicarious trauma” OR “empath* distress” OR “staff wellbeing” OR “moral injury”):ti,ab |
| Emtree | #11 | ‘job stress’/exp OR Burnout/exp |
|  | #12 | (“Compassion* care” OR “patient care” OR “therapeutic relationship”):ti,ab |
|  | #13 | 10 OR 11 OR 12 |
|  |  | 6 AND 9 AND 13 |

**SCOPUS – title/abstract**

| **PICO** | **ID** | **Searches in Title/Abstract** |
| --- | --- | --- |
| Population  #15 Abstract  #16 Title  17 OR | #1 | (Staff OR worker* OR therapist* OR nurse OR nurses OR nursing OR medic* Or doctor* OR psychiatrist* OR teacher* OR psychologist* OR counsellor* OR counselor* OR “social worker*”) |
| Title 18  Abstract 19  OR 20 | #2 | (Ward OR inpatient OR tier 4 OR specialist OR acute care OR “acute treatment” OR admission OR hospitalisation OR hospitalization OR discharge OR PICU OR “psychiatric intensive care unit”) |
| Title 21  Abstract 22  OR 23 | #3 | (“Mental health” OR psychiatr* OR “mental illness” OR CAMHS OR “mental W/3 health” OR “mental W/3 illness” OR “mental ill health” OR “mental W/3 crisis”) |
|  | #4 | 1 AND 2 AND 3 |
| Intervention | #5 | (Intervention* OR training* OR program* OR workbook* OR leadership OR management OR organisational OR “work W/1 conditions” OR prevention OR reduction OR improvement OR strategy OR strategies OR policy OR policies OR “mindfulness W/3 training” OR session OR teach* OR course* OR “quality W/3 improvement” OR Balint OR “schwarz round” OR workload OR caseload OR shift OR supervision OR “reflective practice” OR environment* OR “ward climate” OR “hospital climate” OR team* OR workload OR turnover OR retention OR “shift work” OR culture) |
| Outcome | #6 | (“compassion fatigue” OR burnout OR burn-out OR “secondary trauma” OR STS OR “vicarious trauma” OR “empath* distress” OR “staff wellbeing” OR “moral injury”) |
|  | #7 | (“Compassion* care” OR “patient care” OR “therapeutic relationship”) |
|  | #8 | 6 OR 7 |
|  |  | 4 AND 5 AND 8 |

**BSP**

| **PICO** | **ID** | **Searches in Title/Abstract** |
| --- | --- | --- |
| Population  #15 Abstract  #16 Title  17 OR | #1 | (Staff OR worker* OR therapist* OR nurse OR nurses OR nursing OR medic* Or doctor* OR psychiatrist* OR teacher* OR psychologist* OR counsellor* OR counselor* OR “social worker*”) |
| Title 18  Abstract 19  OR 20 | #2 | (Ward OR inpatient OR tier 4 OR specialist OR acute care OR “acute treatment” OR admission OR hospitalisation OR hospitalization OR discharge OR PICU OR “psychiatric intensive care unit”) |
| Title 21  Abstract 22  OR 23 | #3 | (“Mental health” OR psychiatr* OR “mental illness” OR CAMHS OR “mental N3 health” OR “mental N3 illness” OR “mental ill health” OR “mental N3 crisis”) |
|  | #4 | 17 AND 20 AND 23 |
| Intervention | #5 | (Intervention* OR training* OR program* OR workbook* OR leadership OR management OR organisational OR “work W/1 conditions” OR prevention OR reduction OR improvement OR strategy OR strategies OR policy OR policies OR “mindfulness N3 training” OR session OR teach* OR course* OR “quality N3 improvement” OR Balint OR “schwarz round” OR workload OR caseload OR shift OR supervision OR “reflective practice” OR environment* OR “ward climate” OR “hospital climate” OR team* OR workload OR turnover OR retention OR “shift work” OR culture) |
| Outcome | #6 | (“compassion fatigue” OR burnout OR burn-out OR “secondary trauma” OR STS OR “vicarious trauma” OR “empath* distress” OR “staff wellbeing” OR “moral injury”) |
|  | #7 | (“Compassion* care” OR “patient care” OR “therapeutic relationship”) |
|  | #8 | 6 OR 7 |
|  |  | 4 AND 5 AND 8 |

CINAHL

| **PICO** | **ID** | **Searches in Title/Abstract** |
| --- | --- | --- |
| Population | #1 | Staff OR worker* OR therapist* OR nurse OR nurses OR nursing OR medic* Or doctor* OR psychiatrist* OR teacher* OR psychologist* OR counsellor* OR counselor* OR social worker* |
|  | #2 | Work/ OR personnel, health facility/ |
|  | #3 | 1 OR 2 |
|  | #4 | Ward OR inpatient OR tier 4 OR specialist OR acute care OR acute treatment OR admission OR hospitalisation OR hospitalization OR discharge OR PICU OR “psychiatric intensive care unit” |
|  | #5 | Mental health OR psychiatr* OR mental illness OR CAMHS OR “mental N3 health” OR “mental N3 illness” OR mental ill health OR “mental N3 crisis” |
|  | #6 | 3 AND 4 AND 5 |
| Intervention | #7 | Intervention* OR training* OR program* OR workbook* OR leadership OR management OR organisational OR “work conditions” OR prevention OR reduction OR improvement OR strategy OR strategies OR policy OR policies OR “mindfulness N3 training” OR session OR teach* OR course* OR “quality N3 improvement” OR Balint OR schwarz round OR workload OR caseload OR shift OR supervision OR reflective practice OR environment* OR ward climate OR hospital climate OR team* OR workload OR turnover OR retention OR shift work OR culture |
|  | #8 | Psychosocial intervention/ OR intervention trials/ |
|  | #9 | 7 OR 8 |
| Outcome | #10 | “compassion fatigue” OR burnout OR burn-out OR “secondary trauma” OR STS OR “vicarious trauma” OR “empath* distress” OR staff wellbeing OR moral injury |
|  | #11 | Compassion fatigue/ OR Burnout, professional/ |
|  | #12 | Compassion* care OR patient care OR therapeutic relationship |
|  | #13 | 10 OR 11 OR 12 |
|  | #14 | 6 AND 9 AND 13 |

**Figure S0. PRISMA flow diagram**

**Table S3. Study reports, study types and interventions**

| **Study Report** | **Study Type** | **Intervention** |
| --- | --- | --- |
| Aimola 2018 | Outcomes evaluation (RCT) | Peer-Led Quality Improvement Network |
| Authier 1976 | Outcomes evaluation (RCT) | Micro-counselling training for staff |
| Csipke 2019 | Outcomes evaluation (RCT) | Therapeutic Group Training for Staff |
| Eliassen 2016 | Outcomes Evaluation (non-RCT) | Mindfulness Based Stress Reduction Staff Training and Affect Consciousness Staff Training |
| Ewers 2002 | Outcomes evaluation (RCT) | Psychosocial Ways of Working |
| Forchuk 1998 | Process Evaluation | Transitional Discharge Model (change to ward approach) |
| Forchuk 1998 | Theory Paper | Transitional Discharge Model (change to ward approach) |
| Forchuk 2005 | Economic Evaluation | Transitional Discharge Model (change to ward approach) |
| Forchuk 2007 | Theory Paper | Transitional Discharge Model (change to ward approach) |
| Forchuk 2007 | Process Evaluation | Transitional Discharge Model (change to ward approach) |
| Forchuk 2013 | Process Evaluation | Transitional Discharge Model (change to ward approach) |
| Jasim 2016 | Process Evaluation | Peer-Led Quality Improvement Network |
| McLeod 2006 | Outcomes Evaluation (non-RCT) | Behaviour Modification Skills Training for Staff |
| Moreno-Poyato 2018 | Outcomes Evaluation (non-RCT) | Participatory action research into improving therapeutic relationship |
| Salberg 2022 | Outcomes Evaluation (non-RCT) | Steps towards recovery—a recovery-oriented nursing programme using principles of behavioural activation. |
| Theodoridou 2014 | Outcomes evaluation (RCT) | Model of Integrated Care in Mental Health (change to ward approach) |
| Tyson 2002 | Outcomes Evaluation (non-RCT) | Rebuilding of 2 wards |
| Wykes 2018 | Outcomes evaluation (RCT) | Therapeutic Group Training for Staff |

**Figure S1 ROB2 Ratings for RCTs**


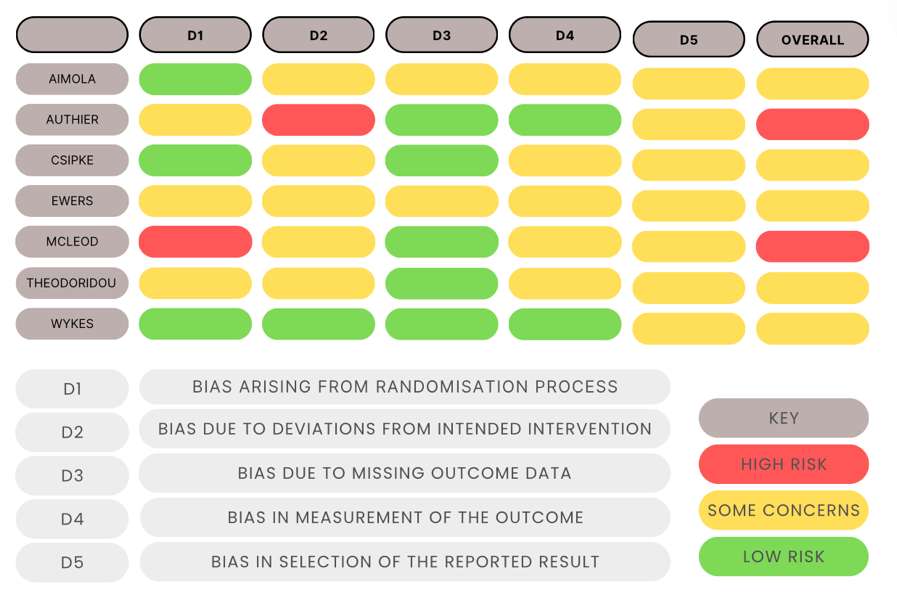


**Figure S2. ROBINS-I Ratings for non-RCTs**


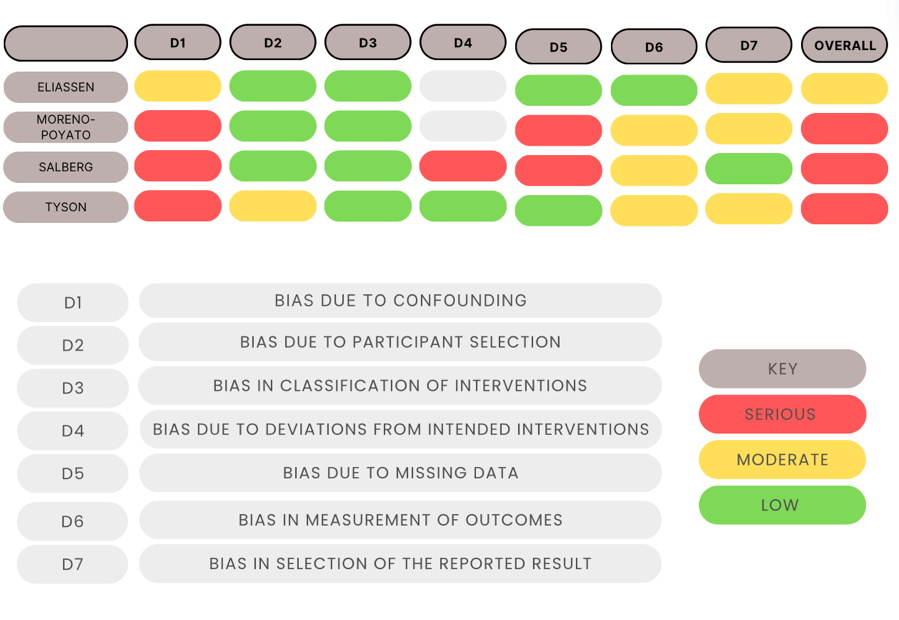


**Table S4. Quality Assessment of Intervention**

| Intervention | Studies | Does intervention refer to a theoretical model? | Is there adequate training for the person delivering the intervention | Has integrity of intervention been checked? | Overall score out of 3 | Effectiveness |
| --- | --- | --- | --- | --- | --- | --- |
|  | Aimola | Yes | Yes/NA | No | 2 | No |
|  | Authier | Yes | Yes | No | 2 | Mixed |
|  | Csipke* | Yes | Yes | No | 2 | No |
|  | Eliassen | Yes | Yes | No | 2 | Yes |
|  | Ewers | Yes | Yes | No | 2 | Yes |
|  | Forchuk 2005^ | Yes | Yes | Yes | 3 | No |
|  | Forchuk 2013^ | Yes | Yes | Yes | 3 | No |
|  | McLeod | Yes | No | No | 1 | No |
|  | Moreno Poyato | Yes | NR | No | 1 | Mixed |
|  | Salberg | Yes | Yes | No | 2 | No |
|  | Theodoridou | Yes | Yes | No N/A | 2 | No |
|  | Tyson | Yes | N/A | N/A | 1 | No |
|  | Wykes* | Yes | Yes | No | 2 | Mixed |

^ and * indicate papers relating to the same intervention.

**S5. Intervention Description Table**

| Intervention | Behaviour Modification Skills Training for Staff (McLeod 2006) | Micro-counselling training for staff (Authier 1975) | Mindfulness Based Stress Reduction Staff Training and  Affect Consciousness Staff Training (Eliassen 2016) | Model of Integrated Care in Mental Health (Theodoridou 2014) | Participatory action research into improving therapeutic relationship (Moreno-Poyato 2018) | Psychosocial Ways of Working (Ewers 2002) | Peer-led quality improvement network (Aimola 2018; Jasim 2016) |
| --- | --- | --- | --- | --- | --- | --- | --- |
| Participants and context *(who is intervention intended for?)* | Mental health nursing staff general acute mental health wards. | Mental health nurses on psychiatric ward of a Veterans Administration Hospital. | Staff on mental health ward which treats “severe mental disorders” sometimes combined with substance use. | Patients from general acute mental health wards | Mental health nurses from acute psychiatric units | Forensic forensic mental health nurses in regional secure units. | Staff and patients on low secure wards. |
| Target problem | Staff burnout | Less beneficial staff-patient interactions (compassionate care) | Variable capacity within staff members to cope with hurtful/violating experiences and to see the patient as someone with the potential to understand and respect. | Lack of interpersonal continuity of care | Poor therapeutic relationship between nurses and patients | Staff lack of knowledge, problematic attitudes and problematic levels of burnout (empathy based stress) | Sub-optimal care (compassionate care) |
| Rationale | Improvement in behaviour modification skills leads to improvement in burnout (via improved self-efficacy and modification of unhelpful attitudes) | Better micro-counselling skills will improve therapeutic relationship between staff and patients | Reflective practice encouraged in staff and enhances understanding of the patient and oneself. Improvement in compassionate care as a result of reflection. | “combination of personal continuity, team continuity and cross-boundary continuity in an acute psychiatric setting improves compassionate care for patient. | Working together with staff group to work out how they think TR and empathy can be improved and implementing site specific changes | Staff having better knowledge and attitudes about treatment for chronic mental illness leads to a greater sense of efficacy and lower burnout | Self-review against consensus standards and independent assessment and feedback improves care quality |
| Socioecological levels targeted | Individual, interpersonal  Community | Individual, intrapersonal  community | Individual, interpersonal,  community | Interpersonal, institutional | Individual, interpersonal, community | Individual, intrapersonal,  community | Interpersonal, institutional, community, |
| IGLOO levels targeted | Individual,  Group | Individual,  Group | Individual  Group | Group  organisation | Individual, group | Individual  Group | Group, leadership, organisational  overarching |
| Materials | None reported | Training course | MBSR: workbook and CD with instructions for home practice  AC: None | None | Journal articles to read plus the nursing best practice guideline “Establishing Therapeutic Relationships” published by the Registered Nurses Association of Ontario (RNAO, 2002) | No physical materials reported | Welcome pack and self-review questionnaires |
| Procedures | Introduction to key behaviour modification concepts e.g. conditional, shaping, modelling, gathering data pre and post treatment, chaining, extinction, modification of behaviours. Homework tasks in between training sessions involving practical application of learning. Videotapes used to assess and develop skills. | Training in original microcounselling skills package plus 4 more complex skills | MBSR: Course developed in accordance with MBSR as described by Kabat-Zinn but reduced time. Included meditation, body scan, yoga, discussion with attitude of openness and acceptance encouraged.  AC: Groups involved teaching and training of affect regulation including 11 basic emotions. Role play of patient-therapist situations and feedback from observers. | Change in practice. One single MDT offer a spectrum of care options from inpatient to community Patients can move in and out of different settings but therapeutic relationships remain consistent. | Nurses asked to compare own practice with that in the reference text. Discussions led to three strategies for improvement. 1) dedicating time each day to patient interaction, 2) reflective staff groups, 3) study of other journal articles.  Staff also used a reflective diary. | Training course in practical skills for reducing distress and improving functioning for people with schizophrenia. A core part of the course was encouraging staff to examine their attitudes and beliefs about psychotic illness. Emphasis on psychosocial model. | Complete peer-review pack, visit from peer reviewers, meeting and feedback report, invitation to forum, access to newsletters and email discussion group. |
| Provider | Clinical psychologist or trainee. | Study authors provided training and supervision | MBSR: The first author of the paper, a trained and experienced MBSR teacher.  AC: A psychologist experienced with affect- consciousness practice. | Staff within mental health teams. Every patient is allocated to the caseload of one physician who is in charge of coordinating all treatments and serves as a primary contact person | Principal investigator lead participatory action research | First author who had training with the Collaboration of Psychosocial Education initiative. Some subject specialists gave guest lectures. | Materials provided by Royal College of Psychiatrists  Peer review visit undertaken by peers from similar wards also in the network |
| Delivery | Face to face training in small groups.  Homework between sessions.  Videotapes used to develop skills in behaviour monitoring and feedback given on development of skills as course progressed. | Face to face or written delivery (depending on group) | Face to face groups for both interventions.  MBSR had additional audio recordings for home practice. | Face to face | Face to face groups to discuss what to do and 2 reflective groups | Face to face. Staff had tso attend 8 sessions, pass a case study submission and pass practical competencies. | Paper based and face to face  Email discussion group  Ward-based intervention |
| Location | Ward | Ward | Ward plus home practice via workbook & CD (MBSR) | Based on a former ward. Care takes place across community and ward settings. | Ward | Ward | Ward |
| Duration | 4 days of training (4 x 6 hour sessions) delivered over 4 week period to small groups of 4 or 5 nurses plus in between session homework tasks involving application of behaviour modification strategies to a patient under their care. | 6 training sessions over 6 weeks. 2 interviews to evaluate training quality  Participants trained with or without supervisor in groups of 6  For supervised group, supervisor meetings were in groups of 3 for a one hour session weekly for 6 weeks | 8 weekly group meetings each lasting 90 minutes, plus 30 minutes of practice and awareness of daily activities.  AC: 8 weekly group meetings each lasting 90 minutes. No home practice expectation. | Sustained from point of admission to discharge. Follow up after 1 year post discharge | Ten month duration of intervention  Unclear how many contacts  2 reflective groups  Daily practice of individual interactions practice  2 articles read | 20 days | Self review takes several weeks, one day of visit, days of time to review other wards, sustained programme. |
| Tailoring | NR | NR | NR | NR | Developed with staff on ward so individualised to that ward | NR | NR |
| Modification | NR | NR | NR | NR | NR | NR | NR |
| Planning | NR | NR | NR | NR | NR | NR | NR |
| Fidelity of adherence | NR | NR | NR | NR | NR | NR | NR |

NR=none reported

Intervention Description Table Cont…

| Intervention | Rebuilding of 2 wards (Tyson 2002) | Steps towards recovery—a recovery-oriented nursing programme using principles of behavioural activation. (Salberg 2022) | Therapeutic Group Training for Staff (Wykes, 2019; Csipke, 2019) | | Transitional Discharge Model (Forchuk, 1998a, 1998b; 2005, 2007a, 2007b, 2013) |
| --- | --- | --- | --- | --- | --- |
| Participants and setting | Staff in psychiatric hospital with general acute and long-stay mental health wards. | Staff on psychiatric inpatient wards (mixture of forensic and general). | Ward staff on general acute psychiatric wards. | Nurses on general acute psychiatric wards. | Staff and patients from psychiatric hospital wards (including forensic, acute and specialised diagnostic wards). |
| Target problem | Iatrogenic ward environment – bad for patients and for staff (and may cause burnout through patient illness being aggravated) | Staff burnout and quality of patient care | Lack of therapeutic activity on wards resulting in poorer perception of care | Low level of staff training in therapeutic interventions and lack of knowledge about the impact of training on staff morale and staff perception of the milieu (empathy based stress) | Lack of continuity of care from hospital to community |
| Rationale | New wards provide more opportunities for interaction and a more pleasant working environment – expected to decrease burnout | Behaviour activation principles improve structure and support for patients which helps both patient care and staff burnout (through improvement of self-efficacy) | Staff skills improved and will benefit patient care | Greater staff satisfaction with care improves staff morale/BO | Continued relationship with staff between inpatient and community services, and provision of a peer support network improves compassionate care |
| Socioecological levels targeted | Institutional | Individual, interpersonal  community | Individual, intrapersonal, | Individual, interpersonal, | Individual  Interpersonal, Institutional, Community |
| IGLOO levels targeted | Organisational | Individual, leadership, group | Individual, group | Individual, group | Individual, leadership, organisational |
| Materials | New buildings | Work sheets and a diary for patients and a manual for nursing staff | Course materials | No physical materials reported | Staff training modules and resources |
| Procedures | Wards were redesigned and rebuilt | 5 steps: engage, explore, ill health & stress, common obstacles & thoughts, plan small steps.  Weekly schedule with daily, 30 minute group sessions each week day using BA principles to help patients make steps towards recovery. Each session has a theme. CBT psychoeducation is part of the approach. Recovery should be seen as overall ward approach too.  4 2 hour training blocks for staff.  Twice monthly meetings for supervision and once monthly meetings for managers. | Four training activities as compulsory as they applied to all wards.   1. (a)  A single session of cognitive–behavioural therapy-based com- munications and understanding/avoiding aggression training for nurses (cofacilitated by a patient educator). 2. (b)  Social cognition and interaction training^25^ aimed at helping people understand social situations better in order to avoid mis- understandings, a common occurrence on wards. 3. (c)  Computerised cognitive remediation therapy (in order to involve occupational therapists), designed to address cognitive deficits such as problems with memory, organisation and concentration.^26^ 4. (d)  Where pharmacists were available, they were recruited to run a medication education group.   Four optional nurse-provided therapies were chosen by wards  based on their patient’s needs (chosen from hearing voices, emotional coping, problem solving, relaxation & sleep, coping with stigma. | Four training activities as compulsory as they applied to all wards.   1. (a)  A single session of cognitive–behavioural therapy-based com- munications and understanding/avoiding aggression training for nurses (cofacilitated by a patient educator). 2. (b)  Social cognition and interaction training^25^ aimed at helping people understand social situations better in order to avoid mis- understandings, a common occurrence on wards. 3. (c)  Computerised cognitive remediation therapy (in order to involve occupational therapists), designed to address cognitive deficits such as problems with memory, organisation and concentration.^26^ 4. (d)  Where pharmacists were available, they were recruited to run a medication education group.   Two optional nurse-provided therapies were chosen by wards  based on their patient’s needs | 2 components:   1. Overlap of inpatient and community staff – inpatient staff continue relationship with clients until clients had a working relationship with a community care provider. 2. Peer support available for 1 year minimum   Staff given 12 hours of training  In subsequent iterations documention systems were developed and designated ward project leaders identified. |
| Provider | Hospital Trust, building contractors | 1-2 members fo staff at each location given role of STR coach and had training to support staff.  All staff had training from experienced nurses.  All coaches had peer supervision meetings and ward managers met once a month. | Following the training workshops the trainer, a clinical psychologist, provided supervision during the intervention period | Clinical psychologists | Ward staff.  Staff were given 12 hours of training in the model.  In subsequent iterations nurses were seconded to the project. |
| Delivery | Ward rebuilt from scratch | Group sessions, face-to-face discussions with patients. Peer supervision for staff, peer supervision for managers. In addition a web based education was designed for new employees. | Face to face | Face to face | Face to face and telephone  In subsequent roll-outs online components were used. |
| Location | Ward | Ward | Ward | Ward | Ward and community  In subsequent iterations off-ward support for documentation systems. |
| Duration | One off building of new wards and move into them. Little detail on length of time this took. | Weekly schedule, 30 minute group sessions every week day  30 minute daily sessions  Training sessions for 2 staff who are coaches, and time to train the rest of the staff  All staff time to take part in training (4 x 2 hour blocks)  Twice monthly meetings for coaches  Ward managers meet once a month  Sustained programme | Workshops in each topic.  Supervision from clinical psychologist for 6 months. | 2-3 hours training per group and weekly modelling for up to 6 months | 12 hours training  Then relationship maintenance - the time for this varied from 0 weeks to 12 months, but the median of the bridging relations between the ward staff and the client was 3 months.  Sustained intervention  In later iterations a weekly teleconference and 3-monthly meetings with researchers were added. |
| Tailoring | NR | NR | Wards chose additional interventions | Wards could choose which optional groups to implement | NR |
| Modification | NR | NR | NR | NR | NR in initial economic evaluation. In process evaluations modifications were described e.g. new topics for training including crisis intervention training, and therapeutic relationship training was combined with boundaries training |
| Planning | NR | NR | NR | NR | NR in economic evaluation. In subsequent studies field notes were kept by the site and submitted monthly. |
| Fidelity of adherence | Building was built to specification | Not measured although user reps and project leader attended regular supervisions | NR | NR | NR for economic evaluation. In subsequent iterations modifications were monitored. |

NR=none reported

**Table S6. Patient and Public Involvement and Effectiveness**

| Intervention | Studies | PPI in design of intervention? | PPI in delivery of intervention? | PPI in evaluation of intervention? | PPI Score (sum of previous three elements) | Effectiveness (from study’s effectiveness results) |
| --- | --- | --- | --- | --- | --- | --- |
|  | Aimola | No | No | No | 0 | No |
|  | Authier | No | No | No | 0 | Mixed |
|  | Csipke 2019 | Yes | ?0 | No? |  | No |
|  | Eliassen | No | No | No | 0 | Yes |
|  | Ewers | No | No | No | 0 | Yes |
|  | Forchuk 2005 - EE | No | No | No | 0 | No |
|  | Forchuk 2007 – theory description | No | No | No | 0 |  |
|  | Forchuk 2013 | Yes | Yes | No | 2 | No |
|  | McLeod | No | No | No | 0 | No |
|  | Moreno-Poyato | No | Yes | No | 1 | Mixed |
|  | Salberg | Yes | Yes | No | 2 | No |
|  | Theodoridou | No | No | No | 0 | No |
|  | Tyson | No | No | No | 0 | No |
|  | Wykes | Yes | Yes | Yes | 3 | Mixed |

Yes=positive effects seen across all outcomes of interest at all time points, Mixed=positive effects across some outcomes of interest across some time points, No=no positive effects seen across outcomes of interest at any time points

**S7. Outcome Effectiveness Table for RCTs**

|  | Peer-led quality improvement network | Micro-counselling training for staff | Therapeutic Group Training for Staff | Psychosocial Ways of Working | Transitional Discharge Model | Behaviour Modification Skills Training for Staff | Model of Integrated Care in Mental Health | Therapeutic Group Training for Staff |
| --- | --- | --- | --- | --- | --- | --- | --- | --- |
| Study | Aimola 2018 | Authier 1976 | Csipke 2019 | Ewers 2002 | Forchuk 2005 | McLeod 2006 | Theodoridou 2014 | Wykes 2018 |
| Target problem | Sub-optimal care (compassionate care) | Less beneficial staff-patient interactions (compassionate care) | Low level of staff training in therapeutic interventions and lack of knowledge about the impact of training on staff morale and staff perception of the milieu (staff burnout) | Staff lack of knowledge, problematic attitudes and problematic levels of burnout (empathy based stress) | Lack of continuity of care from hospital to community | Staff burnout | Lack of interpersonal continuity of care | Lack of therapeutic activity on wards resulting in poorer perception of care |
| Outcome measured | Staff burnout | Therapeutic relationship | Staff burnout | Staff burnout | Quality of life | Staff burnout | Therapeutic relationship | Patient perception of care |
| Is outcome primary or secondary? | Secondary | Primary | Secondary | Primary | Primary | Primary | Secondary | Primary |
| Review domain | Empathy-based stress and compassionate care | Compassionate care | Empathy-based stress | Empathy-based stress | Compassionate care | Empathy-based stress | Compassionate care | Compassionate care |
| Measure used | Maslach Burnout Inventory and P2atient satisfaction with care modified version of the Patient Satisfaction Questionnaire (PSQ) – 4 Qs | Ideal Therapeutic Relationship Scale | Maslach Burnout Inventory | Maslach Burnout Inventory | Quality of Life brief version (QOLI-Brief) | Maslach Burnout Inventory | Scale To Assess Therapeutic Relationships (STAR-D) | Views On Inpatient Care (VOICE) |
| Participants | Staff from 75 low secure wards in England and Wales | 15 registered nurses and 3 licensed practical nurses (LPN) employed on a psychiatric ward of a Veterans Administration Hospital. Participants were female nurses aged 23-61. | 16 general acute wards. Nurses of any grade. 444 took part pre-training measures and 280 in post training measures. | 20 forensic mental health nurses working in a regional secure unit. | Patients from 26 wards in 4 provincial psychiatric hospital in Ontario, Canada. Wards included forensic, acute, specialised for diagnosis wards – paired with similar wards). | Mental health nursing staff in 2 general acute mental health wards. 19 participants and 8 control. | Patients from general acute – 5 wards – 1 integrated the intervention and 4 controls.  178 patients randomised, 93 allocated to intervention | Intervention aimed at staff o general acute psychiatric wards in 5 London boroughs. Measures given to patients. 1108 inpatients took part. |
| Control | Delayed participation in intervention | Control group had no training | Control groups waiting for intervention to be delivered | Wait list control | Control group – treatment as usual | Staff on same wards not undertaking training | 4 units providing standard care – controlled regarding staff, expertise, resources, workloads | Wards waiting for intervention (wards given intervention in waves) |
| Time to follow up from baseline | 12 months | 6 weeks post-training | 6 months (for 48 months) | Pre and post measures, exact timings not specified (but training was 20 days) | Interviews at discharge and follow ups at 1 month, 6 months, 12 months. | Baseline in week before intervention and follow up in week after intervention but exact timing not specified (intervention was 4 days). | Within 24 hours of admission, during initial phase of hospitalisation, upon any change of setting, before discharge, 1 year follow up | 6 months for 48 months |
| Analysis | Burnout analysed by size of the tie by group interaction. Mean difference between groups at follow-up, adjusted for baseline differences at follow-up. MBI analysed as three subscales. | Comparing means and standard deviations pretraining and post training. One between subjects (supervised versus non-supervised versus control) and one within subjects (pretraining versus post training) ANOVA used to analyse Ideal Therapeutic Relationship Scale data. | Means and standard deviations compared pre and post training and effect size of training calculated. | Pre and post scores compared using t-tests | Unidirectional t tests comparing intervention and control groups | Non-parametric tests used to compare between groups and repeated measures analysis of pre/post) | Differences between groups over time analysed with series of generalised linear mixed model (GLMM) | Regression model adjusting for ward and time. |
| Results | Adjusted difference at 12m (95% CI) and p value  Reported as the Network minus Control group  Maslach - Emotional exhaustion scale 1.9 (−0.2, 3.9) p=0.07  Maslach - Depersonalisation scale 1.1 (0.3, 2.0), p=0.007  Maslach - Personal accomplishment scale −0.6 (−2.1, 1.0), p=0.49 | Ideal Therapeutic Relationship Scale ratings of the supervised group were not significantly different from the non-supervised group *(Q* = 1.4S).  The non-supervised group differed significantly from the control group (6 = 4.82, *p <* .OS).  The difference between the supervised group's Ideal Therapeutic Relationship Scale ratings and the control group's Ideal Therapeutic Relationship Scale ratings, although not significant, did approach significance (*Q -* 3.36, *p <* .10)  There was a significant interaction of the supervised group scores over time SS=237.5, df = 2, F=4.89*) | Total MBI score (adjusted for age) training effect effect size (95% CI) =-0.09 (-0.24-0.06), p=0.24  Subscales:  Emotional exhaustion: -0.11 (-0.26-0.04), p=0.16  Depersonalisation  -0.02(-0.19-0.15), p=0.83  Personal accomplishment  0.03 (-0.14-0.19), p=0.73 | No total MBI score given.  Subscale scores:  Emotional exhaustion greater in controls p=0.04  Depersonalisation greater in controls p=0.01  Personal achievement greater in experimental condition p=0.01 | The intervention group did not have a significant improvement in global quality of life (control group mean of 4.65, SD = 1.31; intervention mean = 4.78, SD = 1.31, *F*(1, 22) = 0.38, *P* = 0.27). Similarly the sub-scales were not significantly improved. The exception was that quality of life related to social relations where the specific area targeted by the intervention was improved significantly [*F*(1, 22) = 6.99, *P* = 0.015]. | No significant difference on any subscale of MBI observed for pre and post measures. No p scores reported. | No significant differences between pre and post measures on STAR rating. Staff rating on STAR was the primary measure (Time x integrated case: Beta=0.023 SE=0.021, p=0.285)  No significant change on the patient version of STAR either (Time x integrated case: Beta=0.050, SE=0.043, p=0.254). | Standardised intervention benefit as 0.19 (mean VOICE score pre-intervention = 56.5, S.D. = 19.1, n = 644; mean post-intervention = 54.2, S.D. = 17.2, n = 414).  The only confounder identified was legal status (an a prior moderator) and the adjusted model provides weak evidence for benefit (standardised effect −0.18, 95% CI 0.38 improvement to 0.01 deterioration, p = 0.062).  Two other effects: deterioration on VOICE over time, and voluntary patients more positive about ward environment than involuntary. |

**S8. Outcome Effectiveness for non-RCTs**

|  | Mindfulness Based Stress Reduction Staff Training and  Affect Consciousness Staff Training | Participatory action research into improving therapeutic relationship | Steps towards recovery—a recovery-oriented nursing programme using principles of behavioural activation. | Rebuilding of 2 wards |
| --- | --- | --- | --- | --- |
| Study | Eliassen 2016 | Moreno-Poyato 2018 | Salberg 2022 | Tyson 2002 |
| Target problem | Variable capacity within staff members to cope with hurtful/violating experiences and to see the patient as someone with the potential to understand and respect. | Poor therapeutic relationship between nurses and patients | Staff burnout and quality of patient care | Iatrogenic ward environment – bad for patients and for staff (and may cause burnout through patient illness being aggravated) |
| Outcome measured | How much staff support patients | Therapeutic relationship (compassionate care) | Burnout | Burnout  Also observational data on staff-patient interactions |
| Is outcome primary or secondary? | Secondary | Primary | Primary | Secondary |
| Review domain | Compassionate care | Compassionate care | Empathy based stress and compassionate care | Empathy based stress and compassionate care |
| Measure used | Ward Atmosphere Scale (WAS) (the support subscale relates to compassionate care so those results alone looked at for this review) | Working Alliance Inventory (WAI) | Maslach Burnout Inventory | Maslach Burnout Inventory (MBI)  (Primary is observations of nursing behaviour) |
| Participants | Ward staff in hospital in Norway. 2 wards, with 10 beds and 30 staff members each. At baseline 19 staff members on ward 1 and 17 on ward 2 took part.  Both wards treat patients with “severe mental disorders” sometimes combined with substance use. Ward 1 average length of stay was 8 weeks and ward 2 was 3 weeks | Mental health nurses from acute psychiatric units in two hospitals in Barcelona | Staff on 8 psychiatric inpatient wards. Wards were a mixture of forensic and general. Some sub-specialisms in specific diagnoses. | Staff at a rural psychiatric hospital in Australia. General acute and long-stay mental health wards. 40 nurses on 2 old wards and 40 on 2 new wards. |
| Control | Active control – the other intervention group (but no other control) | Nurses on another ward matched for shift pattern and demographics | Reference group, 3 wards not undertaking the intervention | Old wards compared with new wards |
| Time to follow up from baseline | Two baseline time points (3 months before and 1 week before intervention)  Follow ups at 1 week, 3 months, 6 months and 12 months. | 12 months | 35 months | No detail on specific time period but follow up data gathered approximately 6 months after the ward move |
| Analysis | Paired sample t-tests lookeda t changes in average over time. ANOVA used to test for differences between groups. Also used repeated measures MANOVAS and included only those who had filled in all measur time points. | Comparison of scores before and after intervention using Wilcoxon’s test | Differences within and between groups (STR and reference wards) at baseline and follow up assessed with Chi-square test and Mann-Whitney U-test. Effect sizes calculated using Rosenthal’s r. | Pre and post samples treated as independent samples (due to staff changes) and 2x2 ANOVA used to analyse |
| Results | Significant changes for MBSR group on support subscale scores, between T2 and T3, and T2 and T4. Other subscale scores changed for AC but not related to CC.  MBSR group scores: mean, (SD)  T2 5.8 (0.89)  T3-T2 6.5 (0.86)***  T4-T2 6.4 (0.79)**  T5-T2 6.3 (1.2)  T6-T2 6.4 (0.85)  Cronbach’s Alpha 0.37  AC group scores: M (SD)  T2 6.8 (1.6)  T3-T2 6.9 (1.3)  T4-T2 6.3 (1.2)  T5-T2 6.5 (1.2)  T6-T2 6.3 (1.2)  Cronbach’s alpha 0.71 | Improvements in the total score for the level of therapeutic alliance were statistically significant with a mean difference of more than 7 points between the two groups (p=0.010*).  (Empathy scale measured by Interpersonal Reactivity Index, IRI, also significantly improved with a mean difference of more than 6 points with respect to the comparison group (p=0.026*)) | No statistically significant differences in staff ratings of depersonalisation and personal accomplishment subscales of the MBI. One dimension of MBI decreased on the *control* wards (emotional exhaustion) (p<0.001) with a large effect size (0.50). | Two wards moved into new premises. Burnout increased in both wards on subscales of emotional exhaustion (*F*_1,67_ = 9.57, *P* < 0.01) and personal accomplishment (*F*_1,67_ = 6.57, *P* < 0.05).  (The quality of interactions improved in the acute ward but not in the long stay.) |

**Table S9. PROGRESS PLUS Characteristics Reported and Relevant Subgroup Analyses to Assess Equity Harms**

| Intervention | Studies | PROGRESS-PLUS characteristics reported | Subgroup analysis relating to any PROGRESS characteristics? |
| --- | --- | --- | --- |
|  | Aimola | NR | NR |
|  | Authier | Age, gender, role | NR |
|  | Csipke | Deprivation level, age, gender, ethnicity, English as first language | NR in relation to burnout but in relation to primary outcome (VOTE) a mixed effects regression model did consider potential confoudners of gender, age, ethnicity, employment band, first language and length of employment. |
|  | Eliassen | Gender, age, role, type of employment (staff not patients) | NR |
|  | Ewers | For nurses – language, sex, grade, time at clinic | Demographics compared in groups to check for differences but no subgroup analysis reported for effect. |
|  | Forchuk 2005 | Age, gender, illness | NR but wards matched with reference to illness of patients. |
|  | Forchuk 2007 – theory description |  |  |
|  | Forchuk 2013 | none |  |
|  | McLeod | Age, gender, years of experience | NR |
|  | Moreno Pyato | Sex, experience, age of staff | NR |
|  | Salberg | Age, gender, role | Demographics compared at baseline but no subgroup analysis reported |
|  | Theodoridou | Sex, age, marital status, diagnosis (patients) | NR |
|  | Tyson | Gender and rank | NR |
|  | Wykes | Gender, age, ethnicity | Characteristics compared pre and post and found not to differ. Analysis revealed greater effect on patients under section of MHA. Ethnicity, age, gender subgroup analyses NR |

**S10. Process Table Using Elements of the Context and Implementation of Complex Interventions (CICI) Framework**

| **Intervention** | **Linked studies** | **Theory and rationale** | **Context** | **Implementation** | **Acceptability** |
| --- | --- | --- | --- | --- | --- |
| Peer led quality improvement network | Aimola 2018 | Self-review against consensus standards and independent assessment and feedback improves care quality. | Low secure mental health wards across UK  From Jasim:  Most salient contexts:  Type of external peer review  Length of membership in accreditation scheme  Services with higher baseline readiness for change achieved greater quality improvement through membership of a peer review network (although not statistically significant finding)  Qualitative findings echoed importance of readiness for change constructs. | Ward staff team discuss and complete self review. Peer reviewers come into the ward to review self-ratings and to offer advice and write a report. Self review means staff become aware of practice guidelines. Peer review enables connection with other professionals in similar setting.  Staff turnover was high between time points which impacted intervention.  Key mechanisms of change (from Jasim 2016): sharing & learning, consultation, ownership & delegation, communication, action planning, senior/junior involvement balance. Need sharing and learning, consultation and engagement of senior and junior staff for success to happen.  Most changes occurred before or during self-review and following written feedback. | Little qualitative feedback but authors wondered if staff felt under scrutiny by the process.  Jasim 2016 identified readiness for change as important |
| Micro-counselling programme | Authier 1975 | Imparting skills in micro counselling to help therapeutic relationships | One psychiatric ward of a veterans administration hospital in USA | On the ward small groups either go through written instructions or meet with supervisor for 6 weekly training sessions and 2 interviews. Supervised group were face to face, unsupervised used written instructions.  Teaching of specific skills. Open ended Qs, reflection, Qs into statgements, confrontation, feedback, self-disclosure.  Both groups had some impact but supervisor group was significant change. Importance of personal involvement/ tailoring.  Authors thought 6 hours was too short. | Supervised group reported greater satisfaction (and significant effect). Authors attributed to greater possibility for intervention sessions to flex to supervisee needs. |
| Training staff in delivery of therapeutic groups | Csipke, Wykes | Increasing staff skill improves patient perception of care and staff burnout. | General acute wards in UK. Mix of urban and suburban areas served. Mixed SES. Temporary staff were required to have done at least 7 shifts in the previous month.  Turbulent time on wards. From Csipke: “Three of the five catchment areas in our study reduced the number of wards, and budgets were also reduced. In one ward, staff had to reapply for their own jobs, and some wards did not have ward managers but were run by junior staff or overseen on a part-time basis only.” | Service users involved in design, delivery and evaluation. 4 core trainings and additional modules added on (chosen by staff). Post-training supervision from the trainer. This reduced gradually. 2-3 hours training per group then continued input for at least 6 months. | Staff being able to choose some of the interventions increased staff experience of valuing the interventions and finding them useful and feasible. Very good retention in staff measures for study. |
| Mindfulness Based Stress Reduction Staff Training  Affect Consciousness Staff Training | Eliassen 2016 | Enabling staff to cope with hurtful experiences by being able to acknowledge their own reactions and to treat patinets with understanding and respect. | General wards in a hospital in northern Norway. Both units had 10 beds and 30 staff members. Individualised and supportive therapeutic milieu reported. Also high staff turnover mentioned. | MBSR consists of group meetings and self-practice. Staff take part in mindfulness exercises and reflection. AC groups involved teaching and training of affect consciousness. Both about increasing awareness and tolerating state of mind. | Mindfulness participants were encouraged to practice daily but less than 25% reported practising for 3 times a week or more during the intervention period, and less afterwards |
| Psychosocial intervention | Ewers 2002 | Staff having better knowledge and attitudes about treatment for chronic mental illness leads to a greater sense of efficacy and lower burnout | Forensic wards in UK. Ward staff from one ward were offered intervention | Course delivered over a 20 day period. First author of research paper delivered the intervention in this series of training sessions held on the ward. Content of training was very varied, relating to skill for working with patients in a psychosocial framework. | Only those who volunteered were given the intervention so possibly they were more motivated in the first place. |
| Transitional Discharge Model | Forchuk 1998, 1998, 2005, 2007, 2013 | Greater continuity of care improves experience of compassionate care | Canada – high level of spending on inpatient services. Existing peer support infrastructure. Wards in Canada where intervention developed were very long stay and for patients with schizophrenia.  Staff felt overwhelmed by workplace demands and poorly organised work. Also a context of educational overload with high load of mandatory training.    Scotland, less existing pee support infrastructure and culture. Most peer supporters were female so hard to match. Small study with limited funding.  In Scotland acute wards, mixed presentations.  tertiary care psychiatric hospital sites in Canada including admission wards, schizophrenia wards, mood disorder programs, addictions, dual diagnosis and forensics | Organisational support was key to success. Needed support for change at direct care level, local community level and mental health system level.  Existing infrastructures for peer support made it more likely to succeed.  Organisational change at the same time leading to high turnover had negative impact on success of intervention.  On wards which were slower to take up the intervention the culture was that a lot of meetings to discuss happened but little concrete planning. Change happened when new person came in (eg social worker or new leader)  Lack of role clarity had negative impact on intervention.  If staff were unhappy about other changes they tended to interfere in this one.  Turnover in champions affected intervention especially if at key points.  Changes in clinical environment can impact on intervention working e.g. digital intervention can become ineffective with overuse.  Team issues needed to be addressed prior to implementation  From Forchuk 2007: “Preliminary results highlight the importance of on-ward supports—including champions, easily available teaching modules, and supportive documentation systems. Education sessions need to be paced so that all staff quickly learn the model. Ideally, this requires a couple of half-day sessions or a full-day retreat. Relationships between hospital staff, community agencies, and consumer groups are important for the effective implementation of TDM.”  From Forchuk 2013: Staff were provided with training modules, on-ward champions designated for each ward, and documentation system gradually developed   1. All wards valued having specific education (concentrated interactive workshops) on the TRM prior to implementation. However, the content evolved with each set of wards and the methods of delivery became focused on faster implementation (putting the training modules online) with successive sets of wards. Therefore, the strategies that worked during one period can very quickly become obsolete or unpalatable.’ 2. designated people who led the project whom staff could go to with questions or concerns were recommended by A wards and supported by B and C wards   From Forchuk 1998 (upside down) – key client themes were increased hope and optimism and transition to increased autonomy. Key staff themes were greater satisfaction despite increased demands and importance of relationship/client focus. | Hospital administrators in both Canada and Scotland saw as cost effective and helped with gatekeeping and support.  In Canada liked that it was ‘made in Canada’.  Seen as solution to existing problem  Educational overload and lots of new projects at same time impacted intervention acceptability |
| Skill training in behaviour modification strategies | McLeod 2006 | Improvement in behaviour modification skills leads to improvement in burnout (via improved self-efficacy and modification of unhelpful attitudes) | General acute mental health wards in both Australia and UK | A course on behaviour modification techniques was delivered to staff on the ward. The course included training on the theory and methods, and practical expectations to try the techniques and use video taping to get feedback on their skills. The course was delivered by psychologists and trainee psychologists to groups of 4-5 nurses over 4 weeks (6 hour session each time). Staff had homework in between sessions.  Authors thought the course was too short to have time to take effect, although noted some changes in staff attitude towards patients and behavioural interventions.  Authors also thought the intervention needed to be more multi-faceted and include personal stress management training, enhancement of social support, and ongoing supervision with both skill development and restorative elements. |  |
| Participatory action research into improving therapeutic outcomes | Moreno-Poyato, | Working together with staff group to work out how they think TR and empathy can be improved and implementing site specific changes | Mental health wards in Spain. 2 wards in different hospitals, one with 39 patients and one with 44. Permanent staff from each ward were offered the opportunity to participate. Staff were selected on the basis of profiles created by the researchers to try to capture as many of the different nurse profile as possible. | Nurses were given journal articles and nursing guideline to read and asked to compare their own practice with what they read. Then they were tasked with designing, implementing and assessing strategies to improve therapeutic relationship. The staff chose individual patient care, reflective groups and study of other research. Reflective diaries were also completed (but data not analysed) | During the study 4/9 participants withdrew from the intervention group: 2 for personal reasons, 1 for workload of intervention and 1 for lack of motivation. That is nearly half of group. |
| Steps Towards Recovery (based on behavioural activation) | Salberg 2022 | Behaviour activation principles improve structure and support for patients which helps both patient care and staff burnout (through improvement of self-efficacy) | 8 mental health wards in Sweden. High staff turnover mentioned. Staff at pre and post measures were not the same. | First, 1-2 staff members were given the role of STR coach, did the training then guided and supported other staff.  Second, all staff were given training over 4 2 hour blocks, from experienced nurses.  STR coaches had joint meetings twice a month to share tips, and moanagers met once a month.  Five steps to the training for patients. Daily, 30 minute group sessions each week day were provided by staff for patients. Patients were given worksheets and a diary. Nursing staff were given a manual. | A positive staff experience of the programme was described despite lack of change on outcome measures. Staff turnover was mentioned again as possible limiting factor. |
| Person Centred Integrated Care Model | Theodoridou, 2014 | Improved continuity of people caring for the patient should improve sense of compassionate care. | General acute wards in Switzerland. 5 wards took part – 1 had the intervention. | On one ward practice was changed so that one single multi-disciplinary team offered a range of care options (inpatient, day hospital, outpatient). Each patient allocated one physician in charge of coordinating all treatments and primary contact person. | Authors acknowledge that flexibility of the approach could increase demands on staff members, although they don’t report any differences in staff satisfaction (don’t show specific data). |
| Rebuilding of 2 wards | Tyson, 2002 | New wards provide more opportunities for interaction and a more pleasant working environment – expected to decrease burnout | 2 general acute and long-stay wards in rural Australia with a large catchment area | Wards were redesigned and rebuilt. Staff and patients moved in (little detail on the move or relocation whilst wards being rebuilt) | Interview data describes unintended consequence of intervention. Most staff thought new wards were better for patients but several design flaws were descried, including no rooms for smokers, rooms being too small, and on the acute ward a feeling that it was hard to find other staff members and patients and observations were harder. Nurses reported increased isolation. Authors think organisation needs to respond to physical changes in the ward. |
